# Proteomic analysis identifies key differences in the cardiac interactomes of dystrophin and micro-dystrophin

**DOI:** 10.1093/hmg/ddab133

**Published:** 2021-05-05

**Authors:** Hong Wang, Elena Marrosu, Daniel Brayson, Nalinda B Wasala, Eric K Johnson, Charlotte S Scott, Yongping Yue, Kwan-Leong Hau, Aaron J Trask, Stan C Froehner, Marvin E Adams, Liwen Zhang, Dongsheng Duan, Federica Montanaro

**Affiliations:** Center for Gene Therapy, The Research Institute at Nationwide Children’s Hospital, Columbus OH 43205, USA; Department of Pediatric Cardiology, China Medical University, Liaoning 110004, China; Developmental Neuroscience Research and Teaching Department, Dubowitz Neuromuscular Centre, Molecular Neurosciences Section, UCL Great Ormond Street Institute of Child Health, London WC1N 1EH, UK; NIHR Great Ormond Street Hospital Biomedical Research Centre, London WC1N 1EH, UK; Developmental Neuroscience Research and Teaching Department, Dubowitz Neuromuscular Centre, Molecular Neurosciences Section, UCL Great Ormond Street Institute of Child Health, London WC1N 1EH, UK; NIHR Great Ormond Street Hospital Biomedical Research Centre, London WC1N 1EH, UK; Department of Molecular Microbiology and Immunology, School of Medicine, University of Missouri, Columbia, MO 65211, USA; Center for Gene Therapy, The Research Institute at Nationwide Children’s Hospital, Columbus OH 43205, USA; Developmental Neuroscience Research and Teaching Department, Dubowitz Neuromuscular Centre, Molecular Neurosciences Section, UCL Great Ormond Street Institute of Child Health, London WC1N 1EH, UK; NIHR Great Ormond Street Hospital Biomedical Research Centre, London WC1N 1EH, UK; Department of Molecular Microbiology and Immunology, School of Medicine, University of Missouri, Columbia, MO 65211, USA; Developmental Neuroscience Research and Teaching Department, Dubowitz Neuromuscular Centre, Molecular Neurosciences Section, UCL Great Ormond Street Institute of Child Health, London WC1N 1EH, UK; NIHR Great Ormond Street Hospital Biomedical Research Centre, London WC1N 1EH, UK; Center for Cardiovascular Research, The Research Institute at Nationwide Children's Hospital, Columbus, OH 43205, USA; Department of Pediatrics, The Ohio State University College of Medicine, Columbus, OH 43205, USA; Department of Physiology and Biophysics, University of Washington, Seattle, WA 98195, USA; Department of Physiology and Biophysics, University of Washington, Seattle, WA 98195, USA; Mass Spectrometry and Proteomics Facility, Campus Chemical Instrument Center, The Ohio State University, Columbus, OH 43210, USA; Department of Molecular Microbiology and Immunology, School of Medicine, University of Missouri, Columbia, MO 65211, USA; Department of Neurology, School of Medicine, College of Veterinary Medicine, University of Missouri, Columbia, MO 65211, USA; Department of Bioengineering, College of Veterinary Medicine, University of Missouri, Columbia, MO 65211, USA; Department of Biomedical Sciences, College of Veterinary Medicine, University of Missouri, Columbia, MO 65211, USA; Department of Biomedical, Biological and Chemical Engineering, College of Engineering, University of Missouri, Columbia, MO 65211, USA; Center for Gene Therapy, The Research Institute at Nationwide Children’s Hospital, Columbus OH 43205, USA; Developmental Neuroscience Research and Teaching Department, Dubowitz Neuromuscular Centre, Molecular Neurosciences Section, UCL Great Ormond Street Institute of Child Health, London WC1N 1EH, UK; NIHR Great Ormond Street Hospital Biomedical Research Centre, London WC1N 1EH, UK

## Abstract

ΔR4-R23/ΔCT micro-dystrophin (μDys) is a miniaturized version of dystrophin currently evaluated in a Duchenne muscular dystrophy (DMD) gene therapy trial to treat skeletal and cardiac muscle disease. In pre-clinical studies, μDys efficiently rescues cardiac histopathology, but only partially normalizes cardiac function. To gain insights into factors that may impact the cardiac therapeutic efficacy of μDys, we compared by mass spectrometry the composition of purified dystrophin and μDys protein complexes in the mouse heart. We report that compared to dystrophin, μDys has altered associations with α1- and β2-syntrophins, as well as cavins, a group of caveolae-associated signaling proteins. In particular, we found that membrane localization of cavin-1 and cavin-4 in cardiomyocytes requires dystrophin and is profoundly disrupted in the heart of *mdx^5cv^* mice, a model of DMD. Following cardiac stress/damage, membrane-associated cavin-4 recruits the signaling molecule ERK to caveolae, which activates key cardio-protective responses. Evaluation of ERK signaling revealed a profound inhibition, below physiological baseline, in the *mdx^5cv^* mouse heart. Expression of μDys in *mdx^5cv^* mice prevented the development of cardiac histopathology but did not rescue membrane localization of cavins nor did it normalize ERK signaling. Our study provides the first comparative analysis of purified protein complexes assembled *in vivo* by full-length dystrophin and a therapeutic micro-dystrophin construct. This has revealed disruptions in cavins and ERK signaling that may contribute to DMD cardiomyopathy. This new knowledge is important for ongoing efforts to prevent and treat heart disease in DMD patients.

## Introduction

Patients with Duchenne muscular dystrophy (DMD) lack sufficient expression of a functional dystrophin protein in all striated muscles, leading to loss of ambulation by 13 years of age, progressive respiratory insufficiency and dilated cardiomyopathy (1). Currently, cardiac failure is the leading cause of mortality in DMD with available treatment options having only limited efficacy due to their lack of specificity (2–4). Although the molecular underpinnings of DMD cardiomyopathy are not well understood, gene therapy using micro-dystrophins is emerging as a promising solution (5).

**Figure 1:**
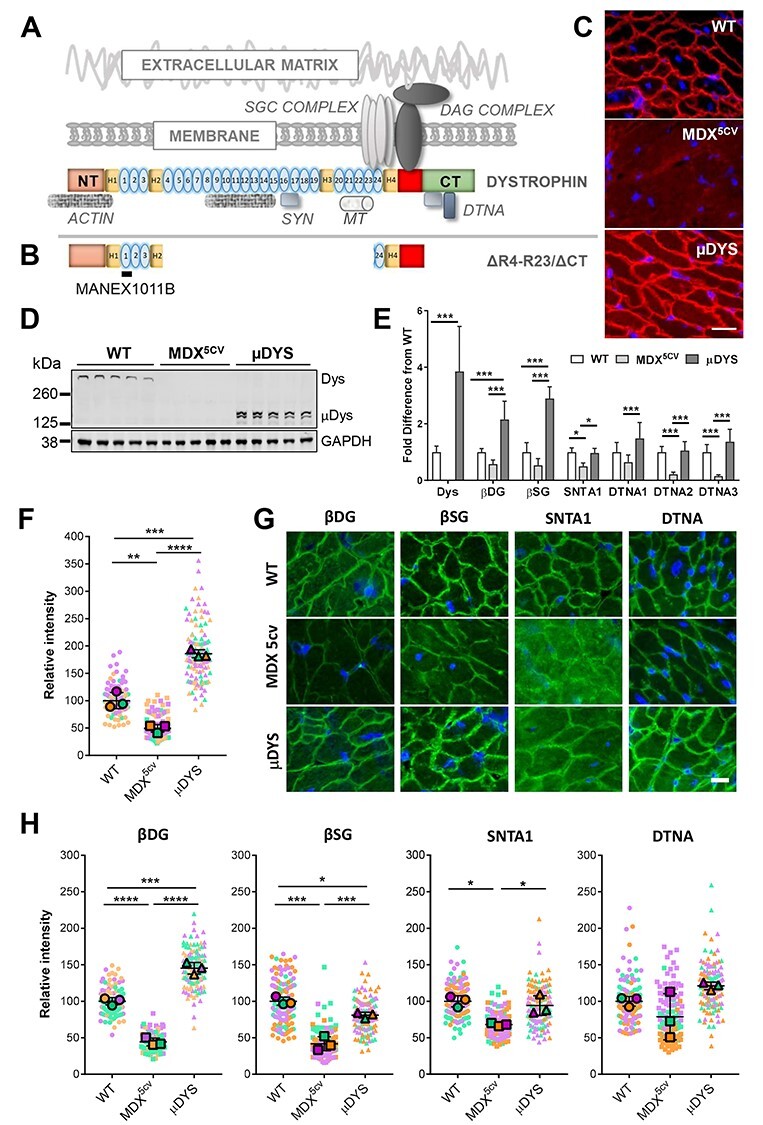
Expression of μDys in cardiomyocytes rescues expression of DAPC proteins. (**A** and **B)** Schematic representation of dystrophin and μDys in relation to each other and to major known protein binding domains. The epitope recognized by the MANEX1011B antibody is marked. DAG: dystroglycans; SGC: sarcoglycans; SYN: syntrophins; DTNA: α-dystrobrevins; MT: microtubules. (**C**) Immunostaining of heart tissue sections from wild-type (WT), *mdx^5cv^* and μDYS-*mdx^5cv^* (μDYS) mice with the MANEX1011B antibody to visualize dystrophin and μDys (red). Scale bar: 20 μm. (**D**) Western blot of total heart protein extracts probed with the MANEX1011B antibody. (**E**) Dystrophin, μDys and DAPC expression levels in lysates from WT (*N* = 5–7), *mdx^5cv^* (*N* = 8–13) and μDYS-*mdx^5cv^* (*N* = 5–6) hearts. Values (mean ± standard deviations) are normalised to wild-type. Corresponding representative western blots are shown in Supplementary Material, Figure S5. ^*^P < 0.05, ^*^^*^^*^P < 0.001, one-way ANOVA. (**F** and **H**) Superplots of immunofluorescence intensity measurements of dystrophin/μDYS and DAPC proteins at the cardiomyocyte membrane. Small symbols are individual immunofluorescence measurements (*N* = 40) per mouse. Large symbols indicate the mean for each individual mouse (*N* = 3/group). Lines indicate the grand mean ± standard deviation. ^*^P < 0.05, ^*^^*^P < 0.01, ^*^^*^^*^P < 0.005, ^*^^*^^*^^*^P < 0.001, two-way repeated measures ANOVA. (**G**) Immunostaining of heart tissue sections with antibodies to the indicated DAPC proteins (green). Nuclei are counterstained with DAPI (blue). Scale bar: 15 μm. βDG: β-dystroglycan; βSG: β-sarcoglycan; SNTA1: α1-syntrophin; DTNA: pan-dystrobrevin antibody; DTNA1: α1-dystrobrevin; DTNA2: α2-dystrobrevin; DTNA3: α3-dystrobrevin.

Micro-dystrophins are miniaturized versions of dystrophin that retain domains essential for bridging the intracellular actin cytoskeleton to the extracellular matrix via the trans-membrane dystrophin-associated protein complex (DAPC; Fig. 1A and B). All micro-dystrophins lack most of the central domain of dystrophin believed to be non-essential for function. This is based on the observation that Becker muscular dystrophy (BMD) patients harboring in-frame deletions in this central domain, produce shorter dystrophin proteins and typically have a mild, late onset disease remaining ambulant for most of their life (6–11). However, the majority of these BMD patients develop severe cardiac disease in their 30’s or 40’s, and cardiac failure remains the primary cause of mortality in BMD (12,13). These clinical observations raise the possibility that current micro-dystrophins may similarly delay but not fully protect from cardiac disease. Of note, pre-clinical studies with ΔR4-R23/ΔCT micro-dystrophin (abbreviated as ‘μDys’ in this manuscript), a micro-dystrophin currently in a phase 1/2a clinical trial (5,14), showed incomplete rescue of cardiac function in the *mdx* mouse model of DMD (15–17). Therefore, there is a need to gain a more detailed molecular understanding of how micro-dystrophins compare to dystrophin in the heart to identify opportunities for further optimization of their cardio-protective efficacy.

The primary role of dystrophin is to organize and stabilize the DAPC (Fig. 1A) at the membrane of muscle cells. Through these protein interactions, dystrophin performs both structural and signaling functions (18,19). We have recently shown that the protein complexes assembled by dystrophin are different in the heart compared to skeletal muscle (20). Specifically, the cardiac DAPC includes additional proteins involved in signaling (β2-syntrophin and α3-dystrobrevin) or important for cardiac function and disease (ahnak1, cypher, αB-crystallin and cavin-1) (20). These findings suggest the existence of cardiac-specific functions of dystrophin that are currently undefined and that may require domains missing in the current design of micro-dystrophins.

To understand how loss of over 60% of the full-length dystrophin protein sequence affects the cardiac DAPC, we characterized the protein complex assembled by μDys in the heart by a combination of functional proteomics, semi-quantitative immunofluorescence and western blot analyses. We report here that full-length dystrophin and μDys assemble protein complexes that differ in their interactions with proteins involved in signaling such as syntrophins and cavins. In particular, we discovered that the cardiac DAPC contains not only cavin-1, as we previously reported (20), but also cavins-2, -3 and -4 indicating a role for dystrophin in caveolae-associated cardiac signaling. Caveolae are small membrane invaginations that facilitate initiation of intra-cellular signaling cascades at the cell membrane important for cardiac physiology and disease (21,22). Within caveolae, cavins are important mediators of cardio-protection, cardiac contraction and cardiac conduction (21,23). Here we show that loss of dystrophin leads to a profound disruption of the membrane localization of two key cavins: cavin-1 and cavin-4. While cavin-1 regulates the formation of caveolae (24,25), the muscle-specific cavin-4 initiates cardio-protective ERK signaling by catecholamines in response to cardiac stress (26). In the adult heart, activation of ERK signaling by catecholamines supports cardiomyocyte survival and induces adaptive hypertrophy to preserve contractile function and force (27,28). We report that ERK activation is suppressed in the dystrophin-deficient heart and that μDys cannot rescue the membrane localization of cavins nor ERK signaling. Our findings reveal a previously unsuspected disruption of cavins and ERK signaling in DMD cardiomyopathy that is not corrected by μDys.

## Results

### Transgenic μDYS-*mdx^5cv^* mice express μDys in cardiomyocytes

To study the μDys-associated protein complex (μDAPC) in the heart, we used transgenic *mdx* mice that express μDys under the control of the cardiac α-myosin heavy chain (αMHC) promoter (MMRRC Stock No: 41194-JAX). For biochemical analyses, we transferred the μDys transgene to the *mdx^5cv^* mouse model (see supplemental materials) because it has lower residual dystrophin expression compared to *mdx* mice (29), rendering it better suited for sensitive proteomic studies.

To confirm expression and correct membrane localization of μDys in the heart of transgene-positive *mdx^5cv^* mice (μDYS-*mdx^5cv^* mice), we performed immunohistochemistry and western blot analyses with the MANEX1011B antibody that recognizes an epitope shared by dystrophin and μDys located close to Hinge 1 of the rod domain (Fig. 1B). A strong and uniform membrane staining was seen in all cardiomyocytes in both wild-type and μDYS-*mdx^5cv^* mice (Fig. 1C; Supplementary Material, Fig. S1). In μDYS-*mdx^5cv^* mice, μDys expression is driven by the cardiomyocyte-specific Myh6 promoter and is therefore restricted to cardiomyocytes. In wild-type mice, the MANEX1011B antibody that recognizes only full-length dystrophin shows immunolabeling of cardiomyocyte membranes only, with no immunoreactivity in interstitial spaces or capillaries that are strongly labelled with an antibody to cavin-1 (30) (Supplementary Material, Fig. S2). By contrast, no staining was detected in the heart of *mdx^5cv^* mice, (Fig. 1C; Supplementary Material, Fig. S1). Quantification of immunofluorescence intensity (31) revealed a 1.7-fold higher expression of μDys above wild-type levels at the lateral membranes of cardiomyocytes (Fig. 1F). Western blot analysis revealed a single protein band at about 430 kDa in wild-type mice, and a doublet at the expected molecular weight (~140 kDa) in μDYS-*mdx^5cv^* mice (Fig. 1D; Supplementary Material, Fig. S3A). This doublet is not detected by the secondary antibody alone (data not shown). Given reports of *in vitro* instability of μDys (32), we suspect that the lower molecular weight band from the doublet is a degradation product of μDys. Accordingly, multiple lower molecular weight bands were detected by the MANEX1011B antibody on full size nitrocellulose membranes in heart lysate samples (Supplementary Material, Fig. S3A) in the absence of general protein degradation as assessed by Ponceau S staining (Supplementary Material, Fig. S3B). No bands were detected in lysates from *mdx^5cv^* mice. Densitometric analysis revealed that μDys (upper band only) is expressed on average 3.9-fold above wild-type levels in μDYS-*mdx^5cv^* heart total protein lysates, with inter-individual differences ranging from 2 to 6-fold (Fig. 1E). Overall, μDYS-*mdx^5cv^* mice express high levels of μDys and the majority of the protein localizes to the cardiomyocyte membrane uniformly across the heart.

## μDys restores expression of DAPC proteins

We next assessed the status of DAPC proteins involved in both structural (β-dystroglycan; β-sarcoglycan) and signaling (α1-sytrophin; α-dystrobrevin) functions. We first analyzed transcript levels by quantitative RT-PCR and found that loss of dystrophin expression in *mdx^5cv^* mice leads to a significant increase in mRNA for dystroglycan, β-sarcoglycan, α1-syntrophin but not α-dystrobrevin relative to wild-type mice (Supplementary Material, Fig. S4). These increases were normalized by μDys expression in μDYS-*mdx^5cv^* hearts (Supplementary Material, Fig. S4). Therefore, loss of dystrophin causes a compensatory up-regulation of mRNA transcripts for some but not all DAPC proteins that is normalized by μDys. We next assessed protein expression levels by western blot analysis. *Mdx^5cv^* mice have a significant reduction in the expression levels of α1-syntrophin, and α-dystrobrevins, and a trend for reduced expression of β-dystroglycan and β-sarcoglycan compared to wild-type mice (Fig. 1E; Supplementary Material, Fig. S5). In μDYS-*mdx^5cv^* hearts, levels of β-dystroglycan and β-sarcoglycan were increased above wild-type levels (>2-fold), while expression of α1-syntrophin and α-dystrobrevins was normalized to wild-type levels (Fig. 1E; Supplementary Material, Fig. S5). By immunohistochemistry and semi-quantitative analysis of membrane fluorescence intensity, *mdx^5cv^* cardiomyocytes showed a significant decrease for all DAPC proteins studied at the cardiomyocyte membrane compared to wild-type, with the exception of α-dystrobrevin (Fig. 1G and H). Additionally, α1-syntrophin consistently showed diffuse intracellular immunofluorescence in *mdx^5cv^* cardiomyocytes (Fig. 1G). In μDYS-*mdx^5cv^* hearts, membrane expression of β-dystroglycan and α1-syntrophin were increased beyond and up to wild-type levels, respectively (Fig. 1G and H) in agreement with western blot quantifications (Fig. 1E). However, diffuse intracellular immunofluorescence was still present for α1-syntrophin in μDYS-*mdx^5cv^* cardiomyocytes. Furthermore, although membrane expression of β-sarcoglycan was significantly increased by μDys compared to *mdx^5cv^* mice, it remained significantly lower than wild-type cardiomyocytes (Fig. 1H) in contrast to our western blot results (Fig. 1E). Overall, μDys normalizes transcript levels, and increases the total protein expression levels and membrane localization of both structural and signaling DAPC proteins in *mdx^5cv^* cardiomyocytes. However, there are differences in the efficacy of rescue at the protein level for different DAPC proteins.

### μDys prevents cardiac histopathology and normalizes electrocardiogram readings

**Figure 2:**
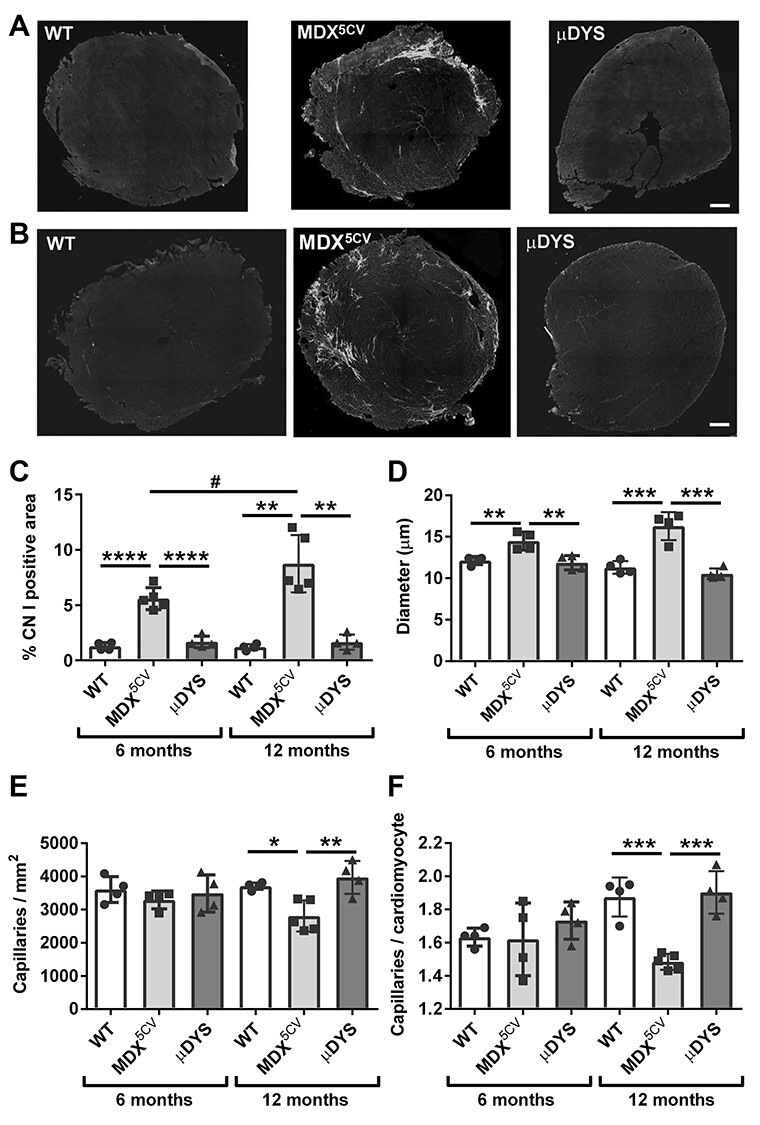
Expression of μDys in cardiomyocytes prevents development of histopathology. (**A** and **B**) Representative montages of heart sections from wild-type (WT), *mdx^5cv^* and μDYS-*mdx^5cv^* (μDYS) mice immunostained for collagen I at 6 months (A) and 12 months (B) of age. Scale bar: 400 μm. (**C**) Quantification of fibrosis based on the percentage of the cardiac section area positive for collagen I. (**D**) Quantification of cardiomyocyte hypertrophy based on measurements of the minimum Feret diameter. (**E** and **F**) Quantification of capillary density normalised to the area (C) or the number of cardiomyocytes (D). Representative images used for quantifications of cardiomyocyte hypertrophy and capillary density are shown in Supplementary Material, Figure S7. Values in graphs are means ± standard deviations. ^*^P < 0.05; ^*^^*^P < 0.01; ^*^^*^^*^P < 0.005, ^*^^*^^*^^*^P < 0.001, one-way ANOVA performed within each age group separately. #*P* < 0.05, Student’s paired *t*-test comparison between 6 and 12 months of age for each individual mouse genotype.

The *mdx^5cv^* mouse model shows late onset cardiac fibrosis and cardiac dysfunction similar to *mdx* mice (33,34). Therefore, we further assessed whether transgenic cardiac-specific expression of μDys prevents the development of cardiac histopathology and improves electrocardiogram readings in *mdx^5cv^* mice. Hematoxylin–eosin staining revealed progressive pathological changes (fibrosis, immune cell infiltration) in heart sections of *mdx^5cv^* mice but not μDYS-*mdx^5cv^* mice between 6 and 12 months of age (Supplementary Material, Fig. S6). In *mdx^5cv^* mice, the area positive for collagen I, a measure of fibrosis, increased from 5.6% ± 0.4 (*n* = 5) at 6 months of age to 8.7% ± 1.2 (*n* = 5) at 1 year of age (*P* value = 0.035, two-tailed unpaired *t*-test; Fig. 2A–C). By contrast, μDYS-*mdx^5cv^* mice did not develop cardiac fibrosis (Fig. 2A–C). Cardiomyocyte hypertrophy and capillary density are affected in heart failure (35) and were quantified in cardiac sections with antibodies to laminin to visualize the basal lamina surrounding capillaries and cardiomyocytes, and CD31 to identify capillaries (Supplementary Material, Fig. S7). Significant cardiomyocyte hypertrophy was present at both 6 months (14.5 μm ± 1.1 versus 12.1 μm ± 0.5 min. Feret diameter; 20% increase) and 12 months of age (16.3 μm ± 1.7 versus 11.3 μm ± 0.8 min. Feret diameter; 44% increase) in *mdx^5cv^* mice relative to wild-type (Fig. 2D). Cardiomyocyte hypertrophy was prevented in μDYS-*mdx^5cv^* mice. Capillary density was significantly decreased in *mdx^5cv^* mice compared to wild-type mice at 1 year but not at 6 months of age (Fig. 2E and F). There was no decrease in capillary density in μDYS-*mdx^5cv^* mice. Electrocardiogram (ECG) evaluation at 6 months of age, revealed significant abnormalities in *mdx^5cv^* mice compared to wild type (prolonged QT interval and QRS duration, lower amplitude and longer duration of the P wave, lower amplitude of the R wave, cardiomyopathy index) that were normalized by μDys (Table 1; Supplementary Material, Fig. S8). Taken together, these results indicate that expression of μDys in cardiomyocytes of *mdx^5cv^* mice recapitulates the disease rescue reported in *mdx* mice treated with a ubiquitously expressed μDys delivered using gene therapy vectors (17,36,37).

**Table 1:** Quantification of electrocardiogram parameters in wild-type, *mdx^5cv^*, and μDYS-*mdx^5cv^* mice at 6 months of age

					*P* value	
Parameter	Unit	WT	Mdx^5cv^	μDYS	*WT* versus *Mdx^5CV^*	*Mdx^5CV^* versus *μDYS*
*N*	–	8	7	5		
HR	bpm	390 ± 29	367 ± 68	419 ± 28	*n.s.*	*n.s.*
P amp.	mV	147 ± 40	99 ± 23	124 ± 19	^*^	*n.s.*
P dur.	ms	24 ± 1	28 ± 2	24 ± 1	^*^ ^*^	^*^ ^*^
PR int.	ms	46 ± 3	42 ± 4	44 ± 1	*n.s.*	*n.s.*
R amp.	mV	800 ± 202	489 ± 114	654 ± 154	^*^ ^*^	*n.s.*
QRS	ms	47 ± 2	62 ± 6	47 ± 2	^*^ ^*^ ^*^	^*^ ^*^ ^*^
QT int.	ms	84 ± 4	104 ± 20	80 ± 4	^*^	^*^
QTc	ms	68 ± 2	80 ± 8	67 ± 3	^*^ ^*^	^*^ ^*^
C.I.	–	3.2 ± 0.5	5.9 ± 0.4	3.4 ± 0.2	^*^ ^*^ ^*^ ^*^	^*^ ^*^ ^*^ ^*^

Data are means ± standard deviation. A one-way ANOVA followed by the Bonferroni test for pair-wise comparisons was performed. No significant differences were found between WT and μDYS-*mdx^5cv^* mice. Amp: amplitude; Dur: duration; Int: Interval; C.I.: Cardiomyopathy Index (QTc/PR segment); *n.s.*: Not significant. Representative traces are shown in Supplementary Material, Figure S8.

^*^ *P* < 0.05

^*^^*^ *P* < 0.01

^*^^*^^*^ *P* < 0.001

^*^^*^^*^^*^ *P* < 0.0001

### Comparison of dystrophin and μDys protein partners in the heart

**Figure 3:**
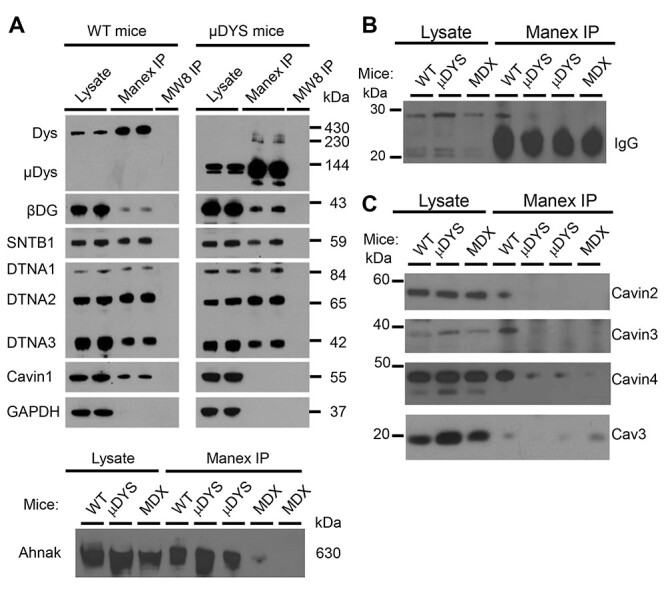
Analysis of proteins that co-IP with dystrophin and μDys. Western blot analyses of total cardiac lysates from wild-type (WT), *mdx^5cv^* (MDX) and μDYS-*mdx^5cv^* (μDYS) mice, and of IPs performed with the MANEX1011B antibody (Manex IP) or the MW8 control antibody (MW8 IP). (**A**) β-dystroglycan (βDG), β1-syntrophin (SNTB1), α1-, α2- and α3- dystrobrevins (DTNA1, DTNA2, DTNA3), and Ahnak are detected in cardiac lysates and MANEX1011B IPs from both wild-type and μDYS-*mdx^5cv^* mice. Full-length cavin-1 is detected in heart lysates from both wild-type and μDYS-*mdx^5cv^* mice but only co-purifies with dystrophin in MANEX1011B IPs. No proteins are detected in control MW8 IPs. (**B**) An antibody to the N-terminus of cavin-1 detects proteolytic fragments of 22 and 28 kDa in all cardiac lysates. The 28 kDa fragment is detected in MANEX1011B IPs (Manex IP) from wild-type (WT) but not *mdx^5cv^* (MDX) or μDYS-*mdx^5cv^* (μDYS) mice. The smaller 21 kDa fragment is obscured by the IgG light chain of the MANEX1011B antibody. (**C**) Cavin-2, -3, and -4 and caveolin-3 (Cav3) are detected in all cardiac lysates. Cavins co-IP with dystrophin in wild-type mice, but are absent or strongly reduced in IPs from μDYS-*mdx^5cv^* mice. Caveolin-3 does not co-IP with either dystrophin or μDys.

We next surveyed the composition of the DAPC and μDAPC in the heart by co-immunoprecipitation (co-IP) using the MANEX1011B monoclonal antibody as previously described (20) (Fig. 3A). Dystrophin and μDys were enriched following IP, but several fainter bands were consistently identified by the MANEX1011B antibody in μDys IPs both above (~230 kDa) and below the expected molecular weight of μDys (Fig. 3A). These bands were not detected in control IPs performed on *mdx^5cv^* cardiac lysates indicating that they are specific. Western blot confirmed successful co-IP of intracellular and transmembrane DAPC proteins with dystrophin and μDys (Fig. 3A). This included co-IP of β1-syntrophin and all three α-dystrobrevin isoforms with both dystrophin and μDys in spite of μDys lacking known binding domains for syntrophins and dystrobrevins (38–42). Furthermore, the cardiac-specific DAPC proteins Ahnak1 and cavin-1 co-purified with dystrophin. However, cavin-1 was undetectable in μDYS-*mdx^5cv^* IPs, suggesting a disrupted association of cavin-1 with μDys (Fig. 3A).

To systematically screen for differentially associated proteins, protein identification by mass spectrometry (MS) was performed on dystrophin IPs from wild-type hearts (*N* = 3) and μDys IPs from μDYS-*mdx^5cv^* hearts (*N* = 3). The μDys sequence was manually added to the peptide search database for accurate peptide matching and was given the ID P11531-A. No peptides matching to domains of dystrophin lacking in μDys were identified in μDYS-*mdx^5cv^* IPs. A total of 121 proteins were identified by MS (Supplementary Material, Table S1). Contaminating/cross-reacting proteins were excluded based on presence in control MANEX1011B IPs on cardiac protein lysates from *mdx^5cv^* mice (*N* = 3) or in a control IP on wild-type lysates with an isotype-matched antibody (MW8; *N* = 1). Utrophin, a homologue of dystrophin, was detected in MANEX1011B IPs indicating some antibody cross-reactivity with utrophin (Supplementary Material, Table S1). However, this contamination was too low to affect our analysis since no DAPC proteins were detected in control IPs. Two proteins we previously reported as part of the cardiac DAPC, cypher and αB-crystallin (20), were identified in control IPs (Supplementary Material, Table S1) and were excluded from further analyses. Immunoglobulins corresponding to the IP antibody were enriched in dystrophin/μDys IPs (Uniprot IDs P01843 and P03987) and were also excluded.

A total of 43 proteins not corresponding to immunoglobulins and never detected in any of our control immunoprecipitations (Supplemetary Material, Table S1) were selected for further analyses (Supplemetary Material, Table S2). These include all previously described cardiac DAPC proteins reported by MS analysis using the MANDYS1 antibody to IP dystrophin (20). Specifically, we confirmed association of cardiac dystrophin with Ahnak1, cavin-1, and β2-syntrophin, as well as lack of association with nNOS. Two additional proteins, cavin-2 and cavin-4, were detected in all dystrophin IPs with high confidence, while cavin-3 was detected in 2 out of 3 dystrophin IPs (Supplemetary Material, Table S2). These cavins are known to interact with each other (43) to regulate the biogenesis, membrane dynamics and signaling of caveolae, small membrane invaginations that perform key physiological functions in the heart (21). Other proteins detected in dystrophin IPs had low total spectral counts and were inconsistently detected. To identify proteins that differentially associate with dystrophin or μDys, we used the exponentially modified protein abundance index (emPAI) as an approximate label-free measure of relative abundance of a given protein between different samples (44). Because dystrophin and μDys are different proteins, their abundance cannot be compared. Protein abundance comparisons were performed between dystrophin and μDys IPs based on emPAI values normalized to the total protein amount in samples (emPAI*^Sample^*) or to the amount of dystrophin/μDys within each sample (emPAI*^DMD^*). Regardless of the normalization method used, α1-syntrophin, cavin-1, cavin-2 and cavin-4 were found to be significantly decreased or absent in μDys IPs compared to dystrophin IPs (Table 2; Supplementary Material, Table S2). Although cavin-3 was not found to be significantly different between dystrophin and μDys IPs (*P* = 0.12), it was not detected in any μDys IP suggesting that its association with μDys might be impaired similar to cavins-1, -2 and -4 (Supplementary Material, Table S2). Using the emPAI*^Sample^* normalization, β2-syntrophin was also found to be significantly decreased in μDys IPs, while BAG3 and tubulin α4A were found to be increased or exclusively detected in μDys IPs. BAG3 interacts with multiple heat shock proteins to mediate re-folding of misfolded proteins or tag them for protein ubiquitination (45,46). This includes Hspb6 and Hspb1 that were detected with 10-fold higher abundance or exclusively in μDys IPs, respectively, compared to dystrophin IPs (Supplementary Material, Table S2). Binding of these chaperone proteins to μDys agrees with the presence of smaller (protein degradation) and higher (protein ubiquitination) molecular weight bands in our western blots of μDYS-*mdx^5cv^* lysates and IPs (Figs 1D, 3A; Supplementary Material, Fig. S3A). Finally, in addition to tubulin α4a, tubulin β2c is also highly enriched in μDys IPs (10-fold; *P* = 0.14; Supplementary Material, Table S2), suggesting a possible preferential association of μDys with microtubules containing tubulin β2c/α4a dimers. Overall, our MS data suggest a decreased abundance of α1- and β2-syntrophins in μDys protein complexes which likely reflects the lack of syntrophin-binding sites in μDys (38–42) (Fig. 1A) and agrees with the partial rescue of membrane localization of α1-syntrophin in μDYS-*mdx^5cv^* cardiomyocytes (Fig. 1G and H). Furthermore, our western blot and MS data indicate a disrupted association of cavin-1 with μDys, as well as novel associations of dystrophin with cavin-2, -3 and -4 that might be impaired with μDys.

**Table 2:** Proteins differentially associated with dystrophin versus μDys

				emPAI*^DMD^*		emPAI *^Sample^*	
Protein	Uniprot ID	Gene Symbol	MW (kDa)	*P* value	FC	*P* value	FC
Syntrophin α1	A2AKD7	Snta1	53	**0.008**	2.7	**0.028**	2.8
Syntrophin β2	Q542S9	Sntb2	56	0.081	7.3	**0.040**	6.5
Cavin-1	O54724	Cavin1	44	**0.003**	5.9	**0.003**	11.0
Cavin-2	Q63918	Cavin2	47	**0.016**	37.5	**0.021**	38.8
Cavin-4	A2AMM0	Cavin4	41	**0.014**	13.0	**0.001**	12.1
BAG3	Q9JLV1	Bag3	62	0.102	0.04	**0.004**	0.1
Tubulin α4A	P68368	Tuba4a	50	0.054	*-INF*	**0.0006**	*-INF*

The emPAI values of the 43 protein that specifically associate with dystrophin/μDYS were normalized to take into account differences in protein amounts between IP samples. EmPAI values for each protein within a sample were either divided by the emPAI of dystrophin/μDys in that same sample (emPAI*^DMD^*) or were normalized to the total number of spectra in each sample using the Scaffold normalization option (emPAI *^Sample^*). A two-tailed Student’s *t*-test was used to identify proteins that differentially associate with dystrophin or μDys (*P* < 0.05; italicized). Fold change (FC) relative to wild-type dystrophin IPs are shown. *–INF* means the protein was only detected in μDys IPs. Complete data are provided in Supplementary Material, Table S2.

### Dystrophin but not μDys associates with multiple cavins

We further investigated the association of dystrophin with cavins and caveolae since caveolae-associated proteins, including cavin-1, cavin-4 and caveolin-3 have been implicated in cardiac disease (21). Furthermore, a link between caveolae and dystrophin in the heart has been previously suggested by a reported interaction of dystrophin with caveolin-3 (47), a muscle-specific caveolar protein. First, we sought to assess why we could detect cavin-1 in μDys IPs by MS but not by western blot. Analysis of the peptides detected by MS revealed that only N-terminal peptides (amino acids 48–97) were detected in μDys IPs while peptides spanning the length of cavin-1 were detected in dystrophin IPs (Supplementary Material, Fig. S9). Since cavin-1 was reported to be proteolytically cleaved in cells (48), we used an antibody recognizing the N-terminus of cavin-1 to identify potential N-terminal proteolytic fragments in cardiac lysates and IPs. By western blot, full-length cavin-1 (55 kDa) and smaller reactive protein bands at ~28 and ~21 kDa in total heart lysates from wild-type, *mdx^5cv^*, and μDys-*mdx^5cv^* mice (Fig. 3A and B). Full-length cavin-1 and the ~28 kDa protein fragment were detected in dystrophin but not μDys IPs. Associations with the ~21 kDa protein fragment could not be ascertained due to overshadowing from the MANEX1011B IgG light chain. Therefore, dystrophin can associate with both full-length cavin-1 and an N-terminal proteolytic fragment of cavin-1, while μDys does not associate with full-length cavin-1 but might bind cavin-1 N-terminal proteolytic fragments at levels too low to detect by western blot. We next assessed whether other cavins are detectable by western blot in dystrophin and/or μDys IPs. Cavin-2, -3 and -4 were detected in cardiac lysates and in dystrophin IPs (Fig. 3C), confirming our MS results. The association with dystrophin is specific since no cavins were detected in control IPs. Furthermore, in agreement with our MS results (Table 2; Supplementary Material, Table S2), cavin-2 and -3 were not present in μDys IPs while cavin-4 was strongly reduced (Fig. 3C). Since caveolin-3 was previously reported to associate with dystrophin in the rat heart (47), we assessed its presence in dystrophin and μDys IPs. However, caveolin-3 was not detected in dystrophin or μDys IPs by either MS or western blot (Supplementary Material, Table S1; Fig. 3C). Taken together, our MS and western blot data indicate that all four cavins associate with dystrophin and these associations are lost or severely disrupted with μDys.

### Dystrophin is required for the membrane localization of cavins but not caveolin-3 in cardiomyocytes

**Figure 4:**
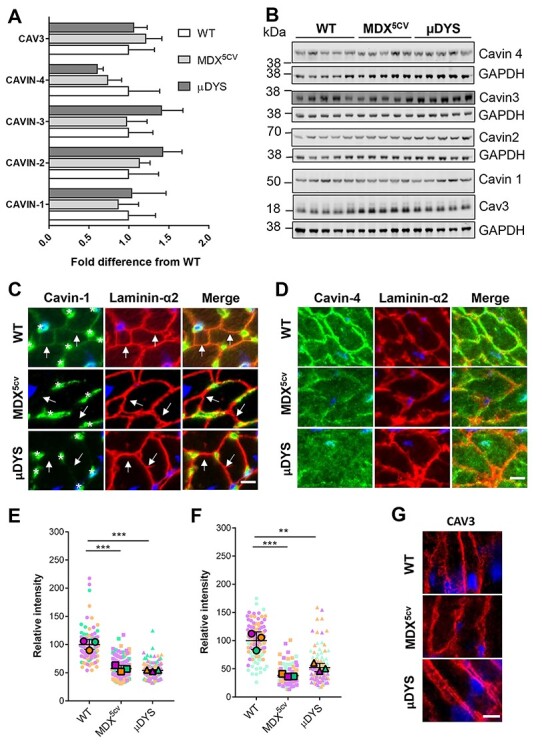
Membrane localization but not expression of cavins is disrupted in *mdx^5cv^* mice and is not rescued by μDys. (**A**) Quantification of protein expression levels of cavins and caveolin-3 (Cav3) in cardiac lysates from 6 months old wild-type (WT), *mdx^5cv^* and μDYS-*mdx^5cv^* (μDYS) mice. Protein levels were normalized to GAPDH probed on the same membrane. Data (mean ± standard deviations; *N* = 5 mice/group) are expressed as fold differences relative to expression levels in wild-type lysates. No significant differences were found (one-way ANOVA). (**B**) Representative western blot used for quantifications shown in A. **C–D**. Immunostaining of heart tissue sections with antibodies to cavins (green) or to laminin-α2 (red) to visualize the membrane of cardiomyocytes and cavin-1 (**C**) or cavin-4 (**D**). Blue = nuclei. In **C**, arrows point to cardiomyocyte membranes to highlight differences in cavin-1 staining between genotypes, while asterisks mark capillaries sitting outside the laminin-α2 outline that are strongly reactive for cavin-1 in all genotypes. **E–F**. Superplots of immunofluorescence intensity measurements of cavin-1 (**E**) and cavin-4 (**F**) at the cardiomyocyte membrane. Small symbols are individual immunofluorescence measurements (*N* = 40) per mouse. Large symbols indicate the mean for each individual mouse (*N* = 3/group). Lines indicate the grand mean ± standard deviation. ^*^^*^p < 0.01, ^*^^*^^*^p < 0.005, two-way repeated measures ANOVA. (**G**) Immunostaining of heart sections for caveolin-3 (red). Nuclei are counterstained with DAPI (blue). Scale bars: 10 μm.

We next assessed the expression of cavins and caveolin-3 in the heart of wild-type, *mdx^5cv^* and μDYS-*mdx^5cv^* mice. Western blot analysis showed no significant changes in protein expression of cavins or caveolin-3 compared to wild-type in either *mdx^5cv^* or μDYS-*mdx^5cv^* mice at either 6 or 12 months of age (Fig. 4C and D; Supplementary Material, Fig. S11A). By immunofluorescence, cavins-1, -2 and -4 all showed continuous staining at the membrane of cardiomyocytes in wild-type mice (Fig. 4C and E, Supplementary Material, Fig. S11A). As previously reported (30), cavin-1 and cavin-2 were also highly expressed in capillaries (asterisks in Fig. 4C, and Supplementary Material, Fig. S11A). In *mdx^5cv^* and μDYS-*mdx^5cv^* mice, cavin-1 was undetectable at the membrane of cardiomyocytes while cavin-2 staining was discontinuous and punctate with increased intracellular staining (Fig. 4C and E, Supplementary Material, Fig. S11A ). Both proteins were preserved in capillaries where neither dystrophin nor μDys are expressed. Cavin-4 was profoundly disrupted with strongly reduced (>2-fold) and discontinuous expression at the cardiomyocyte membrane in both *mdx^5cv^* and μDYS-*mdx^5cv^* mice (Fig. 4D and F, Supplementary Material, Fig. S11B). Unfortunately, the localization of cavin-3 could not be assessed due to lack of a suitable antibody. Caveolin-3 localization at the membrane of cardiomyocytes was indistinguishable between the three genotypes (Fig. 4G). Overall, these results confirm that dystrophin, but not μDys, associates with cavins and further show that dystrophin is required for membrane localization of cavin-1 and cavin-4.

### ERK signaling is disrupted in *mdx^5cv^* hearts and is not normalized by μDys

**Figure 5:**
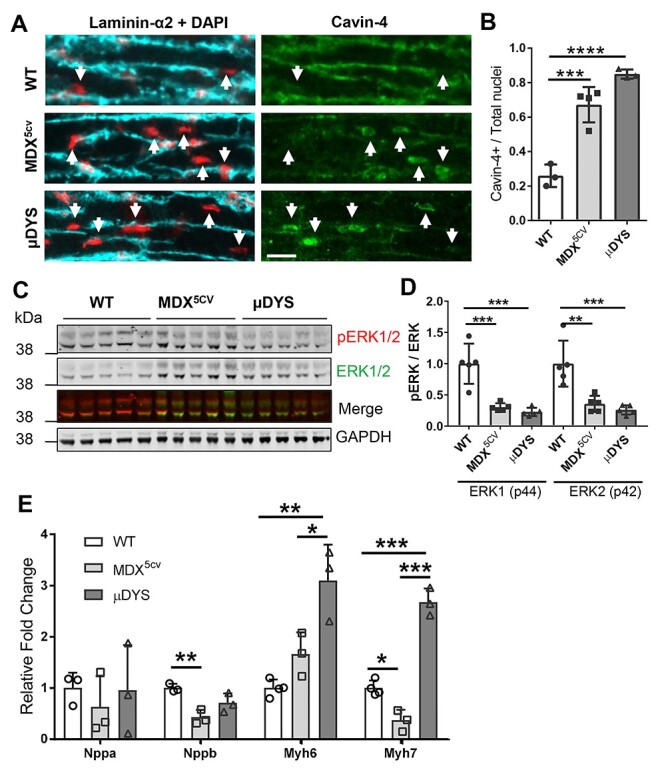
Perinuclear cavin-4 localization is increased and ERK signalling impaired in *mdx^5cv^* and μDYS-*mdx^5cv^* mice. (**A**) Triple staining of heart tissue sections from 6 months old wild-type (WT), *mdx^5cv^* and μDYS-*mdx^5cv^* (μDYS) mice for laminin-α2 (cyan) to visualize the outline of cardiomyocytes, DAPI (red) to visualize nuclei and cavin-4 (green). Arrows indicate nuclei located within cardiomyocytes with perinuclear cavin-4 immunofluorescence. No cavin-4 staining was associated with nuclei from interstitial cells. Scale bar: 25 μm. (**B**) Quantification of cardiomyocyte nuclei with perinuclear cavin-4 labelling relative to the total number of cardiomyocyte nuclei. A minimum of 200 cardiomyocyte nuclei were counted for each mouse. *N* = 3 mice/group. (**C**) Single and double (Merge) fluorescence images of a nitrocellulose membrane double-labelled with antibodies to ERK1/2 (green) and phosphoERK1/2 (pERK1/2; red). The bottom part of the membrane was cut and probed with an antibody to GAPDH to ensure comparable protein loading. (**D**) Densitometric quantification of levels of phosphorylated ERK1/2 (pERK) relative to total ERK1/2 (ERK). *N* = 5 mice/group. (**E**) Quantitative RT-PCR analysis of genes that are regulated by ERK during cardiac remodeling. The ΔΔCt method was used to normalize gene expression to Gapdh. Data was then expressed as a fold-change relative to values in wild-type mice. Data in all graphs are mean ± standard deviation. ^*^P < 0.05; ^*^^*^P < 0.01, ^*^^*^^*^P < 0.005, ^*^^*^^*^^*^P < 0.001, one-way ANOVA followed by a Bonferroni test adjusted for multiple comparisons.

Cavin-4 plays a key role in mediating cardio-protective signaling via the α1-adrenergic receptors (26). Specifically, cavin-4 recruits ERK to caveolae where the latter is phosphorylated following activation of α1-adrenergic receptors by catecholamines. Cavin-4 then translocates with phosphorylated ERK to the nucleus to allow gene activation. ERK phosphorylation via α1-adrenergic receptors occurs when the heart is under stress, and plays an important role in preventing cardiomyocyte apoptosis and activating adaptive cardiomyocyte hypertrophy to preserve contractile strength (27,49). Given the key role of ERK in protecting the heart from injury (50,51) and the reported regulation of ERK signaling by cavin-4 (26), we further investigated the effects of impaired cavin-4 membrane localization on ERK signaling. We postulated that in the presence of cardiac disease in 6-months old *mdx^5cv^* mice, ERK should be phosphorylated and a fraction of cavin-4 should have translocated to nuclei to mediate ERK signaling. In μDYS-*mdx^5cv^* mice where no histopathology is present, ERK phosphorylation should be comparable to wild-type mice and cavin-4 would not be expected to be associated with cardiomyocyte nuclei. We first triple labelled cardiac sections from 6-months old wild-type, *mdx^5cv^* and μDYS-*mdx^5cv^* mice for laminin to see the boundary of cardiomyocytes, DAPI to visualize nuclei and cavin-4 (Fig. 5A). Cavin-4 labelling was associated with 67% and 85% of cardiomyocyte nuclei in *mdx^5cv^* and μDYS-*mdx^5cv^* mice, respectively, compared to 26% of wild-type cardiomyocytes (Fig. 5A and B). We next assessed the status of ERK phosphorylation by western blot (Fig. 5C) and found that phosphorylation of both ERK1 and ERK2 was severely decreased (3 to 4-fold) in the hearts of *mdx^5cv^* and μDYS-*mdx^5cv^* mice compared to wild-type (Fig. 5D). In the context of cardiac overload or damage, activated ERK1/2 phosphorylates the GATA4 transcription factor at Serine 105 which in turn triggers GATA4 binding to its target genes, specifically increasing expression of Nppa, Nppb, and Myh7 while down-regulating Myh6 (52–54). Therefore, if ERK activation is inhibited in *mdx^5cv^* mice then we should observe impaired regulation of GATA4 target genes in spite of the presence of pathological cardiac remodeling. To test this, we quantified mRNA levels of Nppa, Nppb, Myh7 and Myh6 in 1-year-old *mdx^5cv^* mice, when cardiac histopathology and cardiomyocyte hypertrophy are present. We also analyzed μDYS-*mdx^5cv^* mice to determine whether inhibition of ERK signaling below normal baseline levels would alter regulation of these genes even in the absence of cardiac remodeling. Quantitative RT-PCR showed similar levels of expression of Nppa and Myh6 in 1-year-old *mdx^5cv^* and wild-type mice accompanied by a 2-fold down-regulation of Nppb and Myh7 (Fig. 5E). Interestingly, 1-year-old μDYS-*mdx^5cv^* mice showed normal levels of Nppa and Nppb transcripts, but a 3-fold increase in both Myh7 and Myh6 expression compared to wild-type mice. These results indicate that impaired membrane localization of cavin-4 in *mdx^5cv^* and μDYS-*mdx^5cv^* mice is associated with increased cavin-4 peri-nuclear accumulation in cardiomyocytes and with a significant inhibition of ERK phosphorylation below physiological levels. Furthermore, genes specifically regulated by GATA4 downstream of ERK activation are not induced by pathological cardiac remodeling in *mdx^5cv^* mice and the expression of adult and foetal myosin heavy chains is disrupted in μDYS-*mdx^5cv^* mice.

## Discussion

In this study, we have compared the cardiac protein complexes assembled by dystrophin and μDys with the primary goal of identifying protein associations not fully rescued by μDys that might be relevant to cardiac disease/physiology. Unlike prior studies where reconstitution of the DAPC by micro-dystrophins was indirectly inferred by protein co-expression at the cell membrane, we have performed our analyses on purified dystrophin and μDys protein complexes to conclusively ascertain protein associations. In addition, we have quantified the effects of μDys on DAPC mRNA and protein expression not only in total protein lysates by western blot but also at the cell membrane by semi-quantitative immuno-fluorescence (31). The latter technique provides important information on the level of rescue of DAPC proteins at the cell membrane, where they are needed for function. We found no correlation between mRNA and protein expression levels for DAPC proteins indicating that the observed decreases in DAPC proteins in *mdx^5cv^* mice and increases in μDYS-*mdx^5cv^* mice are not regulated at the transcriptional level but at the level of protein translation and/or turnover. Among the DAPC proteins analyzed, only dystroglycan was increased to the same extent as μDys in both western blots and membrane immunofluorescence analyses. We found that large increases (over 3-fold) in protein expression detected in protein lysates by western blot, translate into more modest increases in membrane fluorescence. Furthermore, in the case of cavins, similar protein expression levels were detected by western blot between the genotypes studied, yet membrane localization was profoundly different. Therefore, analyses on total protein lysates on their own are not a good proxy for DAPC protein expression at the membrane or for identification of dystrophin-associated proteins. Overall, our study shows that MS, immuno-precipitation and quantification of protein membrane fluorescence provide important additional information when evaluating DAPC rescue by micro-dystrophin constructs developed for gene therapy. While the approach we used is ideal to rapidly identify and validate differential protein associations, additional complementary biochemical approaches should be considered to explore further the differences we found between the cardiac DAPC and μDAPC. Among these, approaches previously used to study the assembly, composition and stoichiometry of the DAPC (55,56) could be applied to determine whether discrepancies between total protein levels and membrane expression of some DAPC proteins observed in our study occur at the level of the Golgi/ER or the plasma membrane, and to provide additional information on a number of interesting parameters such as the strength of protein–protein interactions, the composition and stability of protein sub-complexes within the μDAPC relative to the DAPC.

Our study shows that μDys is able to assemble a cardiac protein complex very similar to dystrophin that includes the cardiac-specific DAPC protein Ahnak1 (20) but shows an impaired association with cavins (discussed below). We also report that the μDAPC includes the full complement of syntrophins (α1, β1 and β2) and all three α-dystrobrevin isoforms, in spite of μDys lacking all known binding domains for syntrophins and dystrobrevins (38–42). This finding agrees with prior immuno-histochemical studies showing restoration of α1-syntrophin and dystrobrevins at the membrane of skeletal muscle fibers expressing micro-dystrophins (57,58). These associations are likely mediated via sarcoglycans that bind α-dystrobrevins which in turn bind to syntrophins (59,60). However, our quantitative MS analyses on purified protein complexes indicate that the μDAPC contains reduced levels of α1- and β2-syntrophins. This is further supported by our western blot and membrane immunofluorescence quantifications indicating that although μDys is expressed at levels greater than 2-fold above wild-type, α1-syntrophin and α-dystrobrevin levels are not increased accordingly. These results indicate that the loss of syntrophin and dystrobrevin binding sites in μDys does have an impact on the stoichiometry of the μDAPC. In skeletal muscle, neuronal nitric oxide synthase (nNOS) exclusively associates with the DAPC when α1-syntrophin binds directly to dystrophin (41,61). Therefore, the mode of association of syntrophins with dystrophin and micro-dystrophins can impact the composition and function of the DAPC. While nNOS is not part of the cardiac DAPC (20), it remains to be ascertained whether cavins may similarly bind to a syntrophin directly bound to dystrophin, thus explaining their severely impaired association with μDys. Overall, our results indicate that there are hitherto unrecognized quantitative differences in syntrophins, dystrobrevins and cavins between the protein complexes assembled by dystrophin and μDys in the heart.

Our study further revealed a subset of protein associations preferentially involving μDys. Detection of higher levels of BAG3 and its interacting proteins Hspb1 and Hspb6 in μDys IPs agrees with prior reports of aggregation and instability of μDys *in vitro* (32). The occurrence of μDys degradation and ubiquitination *in vivo* are further supported by our western blot analyses showing additional specific bands at both lower and higher molecular weights in μDYS-*mdx^5cv^* cardiac extracts. The selective association of μDys with tubulin α4a is intriguing. μDys lacks the microtubule binding domain located within spectrin repeats R20-R23 (Fig. 1A and B) and does not bind microtubules *in vitro* (62). However, *in vivo* μDys co-sediments with α-tubulin (63) and improves, but does not fully normalize, the organization of the tubulin lattice in *mdx* mice (64). Our MS results suggest that μDys might associate with tubulin α4a in a dimer with tubulin β2c, an unexpected result that needs further investigation.

The major finding of this study is a new link between cardiac dystrophin and all four known cavins. This link is supported by our MS and western blot analyses of purified DAPCs, as well as our immuno-histochemical analyses on tissue sections. Cavins are important players in cardiac physiology and disease primarily via their ability to control caveolar dynamics and signaling. In particular, cavin-4 is emerging as a mediator of several signaling cascades in the heart including calcium homeostasis and cardio-protection (26,65,66) while cavin-1 is essential for the formation of caveolae in cardiomyocytes (24). Therefore, our novel finding that membrane expression of cavin-1 and cavin-4 is severely decreased in dystrophin-deficient cardiomyocytes suggests that one or more caveolar functions affecting cardio-protection, cellular homeostasis, cardiac contraction and/or conduction might be impaired in DMD cardiomyopathy (21,23). While we could not confirm a previously reported interaction of dystrophin with caveolin-3 (47), the major caveolar protein in cardiac cells, a functional link between dystrophin and caveolae is supported by our finding that ERK signaling, a pathway known to be in part regulated via caveolae and cavin-4 (26), is impaired in *mdx^5cv^* mice. This disruption of ERK signaling is not secondary to the presence of cardiac pathology for three reasons. First, ERK phosphorylation is typically induced, not suppressed by cardiac stress/damage (67) and is required for changes in the expression of cardiac hypertrophy genes regulated by the GATA4 transcription factor (52–54). Therefore, we would expect to see a strong activation of ERK and correlated changes in gene expression of Nppa, Nppb, Myh7 and Myh6 in the hearts of *mdx^5cv^* mice at 6 months and 1 year of age. Second, cardiac histopathology is rescued by μDys in μDYS-*mdx^5cv^* mice yet ERK phosphorylation is not normalized and expression of two genes it regulates, Myh6 and Myh7, is affected. Third, since μDys rescues all DAPC proteins except cavins, our findings in μDYS-*mdx^5cv^* mice strongly implicate dystrophin as a direct regulator of ERK signaling via cavins.

A direct regulation of ERK by dystrophin implies that ERK signaling is impaired early in the DMD heart and is likely an important contributor to cardiac disease progression in DMD. An early defect in ERK signaling could potentially affect post-natal cardiac development/maturation. Our finding that μDYS-*mdx^5cv^* mice highly co-express Myh6 and Myh7 could indicate a defective replacement of Myh7 by Myh6 that normally occurs by 7 days after birth in mice (68). While these are target genes of ERK during adult cardiac remodeling, it is not known whether ERK is involved in their early post-natal regulation. It will be interesting to determine whether this developmental switch is affected in young *mdx^5cv^* mice and in μDYS-*mdx^5cv^* mice leading to co-expression of Myh6 and Myh7, which could have an impact on cardiac contractility since these myosins have different energy requirements and cross-bridging dynamics (69). This knowledge could be relevant to the timing of gene therapy interventions but would also suggest the presence of very early changes in myofibrillar protein composition in DMD and BMD hearts. Based on the known functions of ERK in adult cardiac remodeling, we anticipate that impaired ERK signaling will have an important impact upon two main features of cardiac disease in DMD: cardiomyocyte death and adaptive hypertrophy. Prior studies have shown that ERK inhibits cardiomyocyte apoptosis and that decreased ERK phosphorylation leads to increased cardiomyocyte apoptotic cell death in response to damage (50,51,67). Therefore, sub-basal levels of ERK phosphorylation in dystrophin-deficient cardiomyocytes are predicted to increase susceptibility to cell death. A second well documented cardio-protective function of ERK activation is induction of cardiomyocyte hypertrophy (28). Cardiomyocyte hypertrophy can occur when ERK phosphorylation is absent or reduced (51), and we did observe cardiomyocyte hypertrophy in 6-months and 1-year old *mdx^5cv^* mice without activation of hypertrophy genes controlled by ERK via its action on the transcription factor GATA4 (52–54). While multiple signaling pathways can induce cardiomyocyte hypertrophy, there is a qualitative difference: ERK induces a protective form of hypertrophy known as adaptive hypertrophy that preserves cardiac function and contractility protecting the heart from further damage (28,53,67,70). Of particular relevance, cavin-4 has also been implicated in activation of adaptive hypertrophy by facilitating ERK activation downstream of the cardiac α1-adrenergic receptors (26). It is tempting to speculate that by anchoring cavin-4 to the cardiomyocyte membrane, dystrophin facilitates activation of ERK by the α1-adrenergic receptor to induce adaptive hypertrophy and protect the heart from cardiac damage (20,27,71). Therefore, our findings suggest very concrete new avenues of research into a new molecular link between dystrophin and activation of cardio-protective mechanisms that are relevant to DMD cardiomyopathy, and more generally to physiological and pathological cardiac remodeling.

Finally, our finding that μDys does not rescue cavin-1 and cavin-4 membrane localization or ERK phosphorylation identifies specific biochemical deficits of this gene therapy construct in the heart. It is important to emphasize that although μDys prevents cardiac fibrosis, normalizes cardiomyocyte size, corrects electrocardiogram abnormalities, and improves cardiac function in dystrophin-deficient mice as reported by us and Townsend *et al.* (17), these findings do not imply that impaired ERK1/2 signaling is of no concern. Purcell *et al.* (51) have characterized DUSP6 mice with a selective inhibition of ERK1/2 phosphorylation in the heart. Like μDys-expressing *mdx* mice, DUSP6 mice have a normal life span, do not develop cardiac fibrosis, have normal cardiac function, and their cardiomyocyte size is similar to wild-type mice. However, when DUSP6 mice are challenged with cardiac overload, they show enhanced cardiac fibrosis, inflammation, chronic cardiomyocyte apoptosis, and cardiac decompensation. Therefore, the implications of impaired cardiac ERK1/2 signaling become manifest only under conditions of stress. In addition to ERK disruption, μDys expressing hearts likely suffer from additional deficiencies related to loss of cavin-1 expression at the membrane of cardiomyocytes. In particular, caveolae and cavin-1 play an important role in membrane repair in muscle cells (72) and cavin-1 associates with the membrane-repair proteins dysferlin (73) and MG53 (74). Mice lacking MG53 or dysferlin do not show overt heart disease at baseline but are vulnerable to cardiac dysfunction under stress conditions (75,76). While μDys protects *mdx* mice from immediate death following acute dobutamine-induced cardiac stress (17), to date no studies have assessed the ability of micro-dystrophins to support the long-term recovery of the heart following acute stress or prolonged exposure to chronic physiological stressors. Our results indicate that such investigations are needed. Results would be highly informative not only for micro-dystrophin gene therapy but to understand risk factors that may trigger cardiac disease in BMD patients expressing internally deleted dystrophins with impaired associations with cavins.

Overall, our findings suggest new avenues of research into the role of dystrophin in cardiac remodeling in general and more specifically into the molecular underpinnings of cardiac disease in DMD and BMD patients. We have provided a more detailed characterization of the protein complex assembled by μDys in the heart. Our findings point to possible limitations of μDys in terms of its long-term cardioprotective efficacy, in particular in the presence of heart stressors that can be experimentally tested. Identification of the domain(s) of dystrophin required for association with cavins could help guide the design of future micro-dystrophins with improved cardiac protection, and to inform future exon skipping or gene editing strategies aimed at the heart.

## Materials and Methods

Detailed methods are provided in the supplemental material.

### Animals

Animal breeding and experimental procedures followed approved protocols by the Institutional Animal Care and Use Committees at Nationwide Children’s Hospital and University of Missouri.

### Purification and analysis of dystrophin and μDys protein complexes

Purification and MS analyses of complexes assembled by dystrophin (DAPC) and μDys (μDAPC) were performed as previously described (20) using the MANEX1011B monoclonal antibody that recognizes both full-length dystrophin and μDys (Fig. 1A and B). Mascot (Matrix Science) and Scaffold (Proteome Software, Inc.) were used for data analysis. Confidence thresholds were set at 95% for both peptides and protein identifications, and only proteins identified by a minimum of three peptides in any sample were considered as valid identification. Label free quantitation was performed using the emPAI defined as 10^PAI^-1, where PAI (Protein Abundance Index) denotes the ratio of observed to observable peptides for a given protein. For quantitative analyses, the emPAI was normalized either to the protein amount in each sample using the normalization function in Scaffold, or divided by the emPAI of dystrophin/μDys within the same sample.

### Statistical analyses

The Shapiro–Wilk normality test was performed. Normally distributed data were analyzed using a two-tailed Student’s *t*-test or a one-way ANOVA followed by a Bonferroni test for pair-wise comparisons with a *P* value set at 0.05 and with correction for multiple comparisons. Non-normally distributed data were analyzed using the non-parametric Kruskal-Wallis analysis followed by the Dunn’s test for pair-wise comparisons. A two-way repeated measure ANOVA was used for superplots involving nested measurements of membrane fluorescence intensity for *n* = 3 mice per group.

## Supplementary Material

Wang_et_al_Suppl_Figures_revised_ddab133

Wang_et_al_Supplemental_Tables_ddab133

SUPPLEMENTAL_MATERIAL_Revised_Final_ddab133
