# Supplementary material for: Proteomic analysis identifies key differences in the cardiac interactomes of dystrophin and micro-dystrophin
Source: Hum Mol Genet. 2021 May 5;30(14):1321–36. doi: 10.1093/hmg/ddab133 (PMC8255133; doi:10.1093/hmg/ddab133)
Supplement: Wang_et_al_Suppl_Figures_revised_ddab133 [file wang_et_al_suppl_figures_revised_ddab133.pdf]

Figure S1: μDYS is uniformly expressed in the myocardium of transgenic μDYS-*mdx*^{5cv} mice up to 1 year of age. Montages of cardiac sections from 1-year old wild type (WT), *mdx*^{5cv} (MDX) and μDYS-*mdx*^{5cv} (μDYS) mice stained with the MANEX1011B antibody (red) that recognizes an epitope present in both full length and micro-dystrophin.

Figure S1

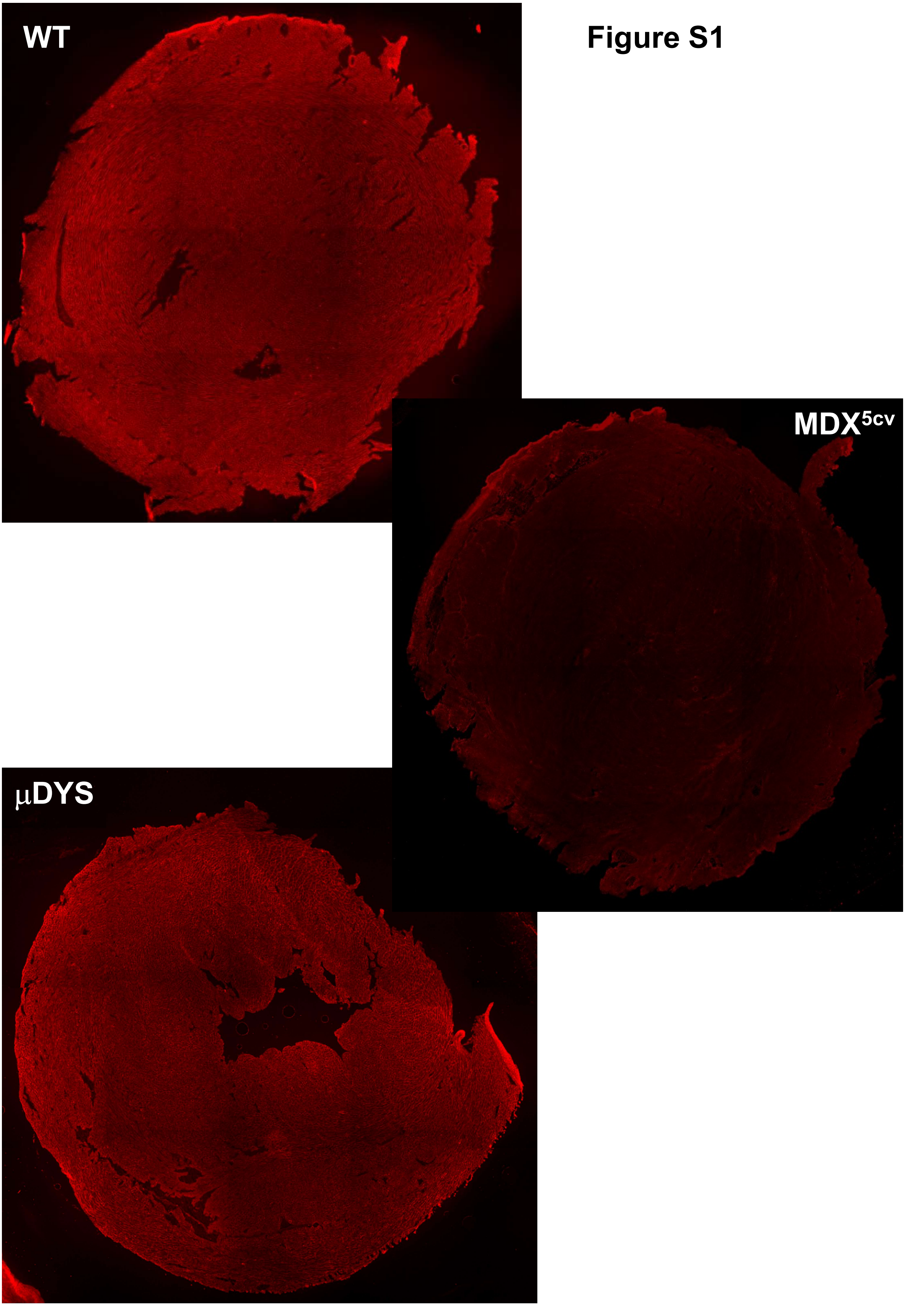

Figure S2

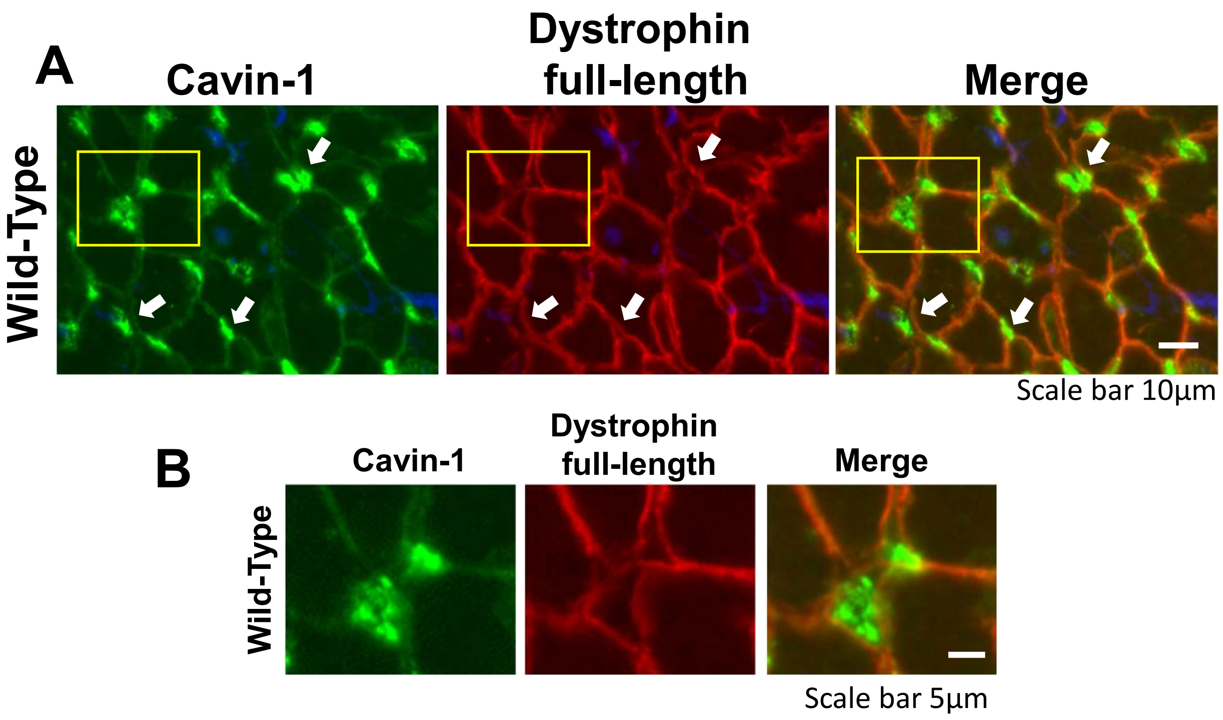

Figure S2: Full length dystrophin is expressed in cardiomyocytes and is absent from capillaries. A. Representative images of cardiac sections from a 6 months old wild-type male mouse double-labelled with the MANEX1011B antibody (red) that only recognizes full-length dystrophin, and with an antibody to cavin-1 (green) a protein highly expressed in capillaries in the mouse myocardium. Scale bar = 10µm. **B.** Higher magnification of the boxed area in A to highlight the lack of dystrophin (red) expression in capillaries that are highly positive for cavin-1 (green) and in the interstitial tissue in-between cardiomyocytes. Note that cavin-1 is also expressed at lower levels at the membrane of cardiomyocytes where it co-localizes with dystrophin. Scale bar = 5µm.

Figure S3

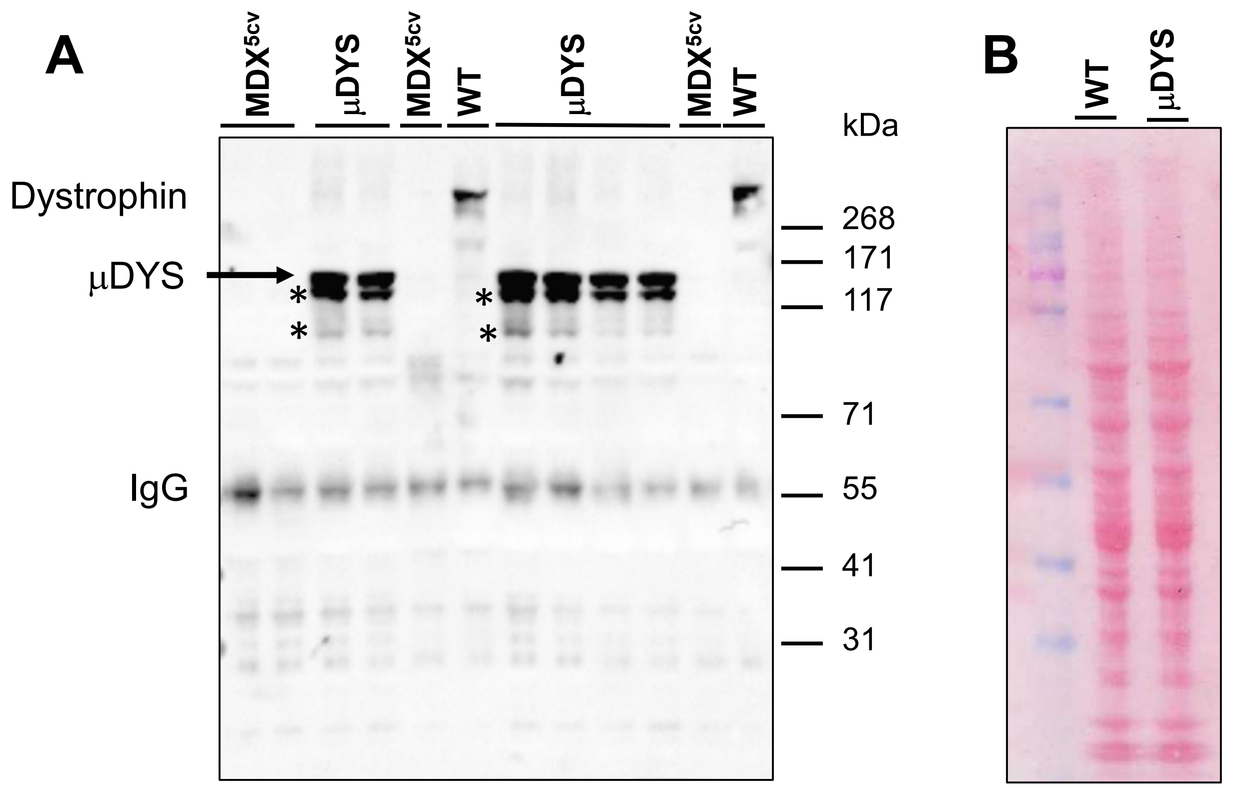

Figure S3: µDYS stability *in vivo*. A. Full nitrocellulose membrane of cardiac lysates from wild type (WT), *mdx*^{5cv} (MDX) and µDYS-*mdx*^{5cv} (µDYS) mice probed with the MANEX1011B antibody. The image was over-exposed to reveal the presence of additional lower bands (asterisks) and a smear below the 144 kDa µDys band (arrow) in the µDYS-*mdx*^{5cv} samples. **B.** Representative nitrocellulose membrane stained with Ponceau S to visualize all proteins in heart lysates from a wild-type and a µDYS-*mdx*^{5cv} mouse. Both samples show well-defined protein bands and no smears indicating a lack of global protein degradation.

Figure S4

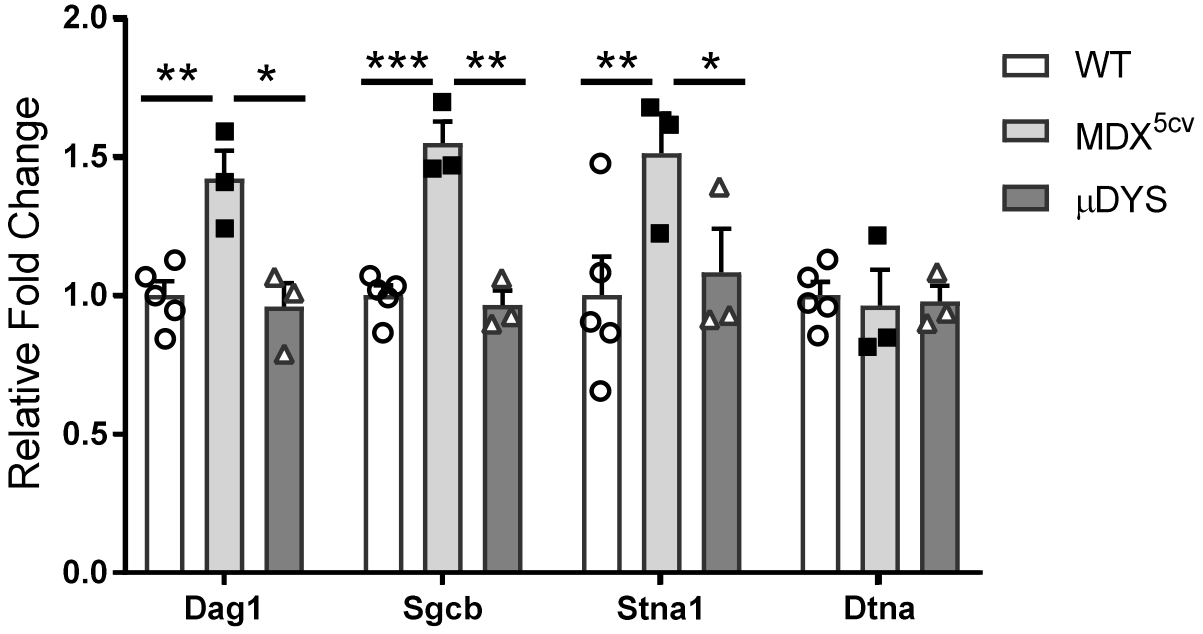

Figure S4: Quantification of DAPC mRNA transcripts in hearts from wild-type (WT), *mdx^{5cv}* and µDYS-*mdx^{5cv}* (µDYS) mice. Transcript amounts for the indicated genes were quantified by quantitative RT-PCR using the ΔΔCt method. Transcript levels were normalized to HPRT and then further normalized to levels in wild-type samples. Statistical differences were determined using an ordinary two-way ANOVA followed by the Tuckey test with correction for multiple comparisons. * $p<0.05$; ** $p<0.01$; *** $p<0.005$. DAG1: dystroglycan; SGCB: β-sarcoglycan; STNA1: α1-syntrophin; DTNA: α-dystrobrevin.

Figure S5

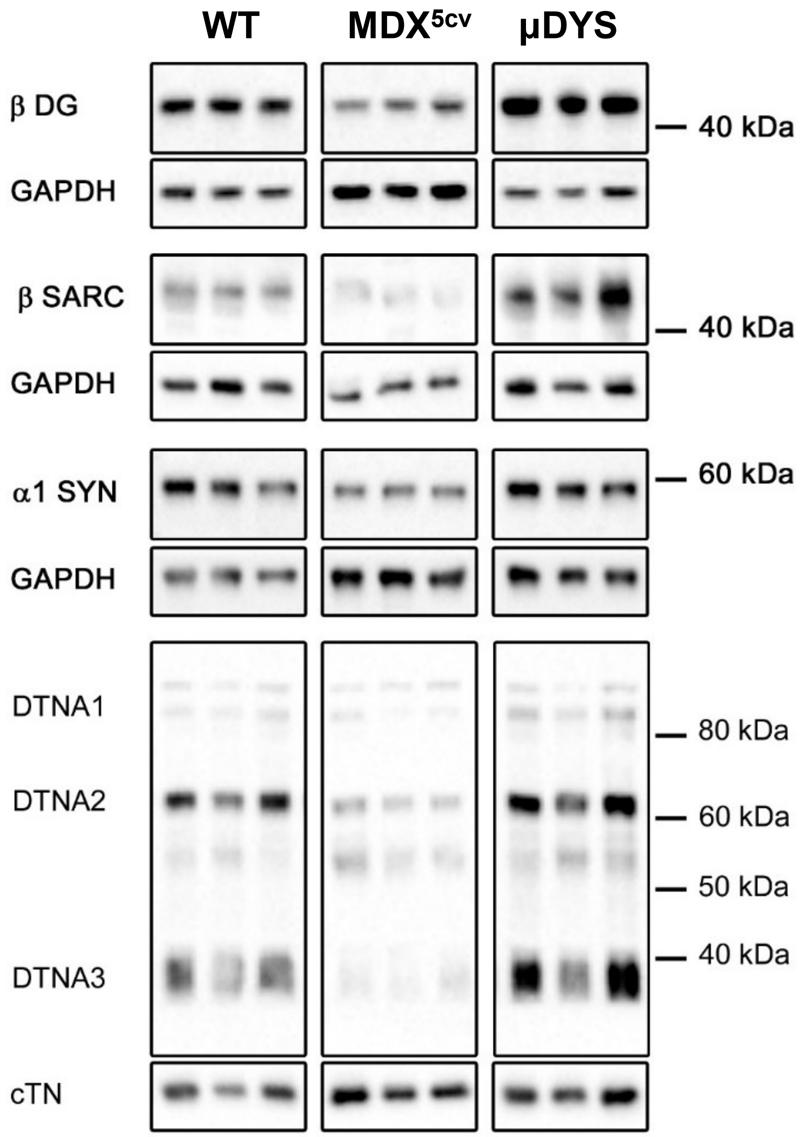

Figure S5: Representative western blots of total cardiac lysates from wild-type (WT), *mdx^{5cv}* and µDYS-*mdx^{5cv}* (µDYS) mice probed for DAPC proteins. After transfer, nitrocellulose membranes were cut at appropriate molecular weights to allow probing for multiple proteins, including loading controls (GAPDH or cardiac troponin (cTN) depending on the protein molecular weight) on the same gel. Each membrane had 3-4 samples corresponding to individual mice for each genotype. Densitometry was performed using a Chemidoc apparatus and intensity of bands for the proteins of interest was first normalized to the loading control on that exact same sample, and then to the reference wild-type samples on the same membrane.

Figure S6

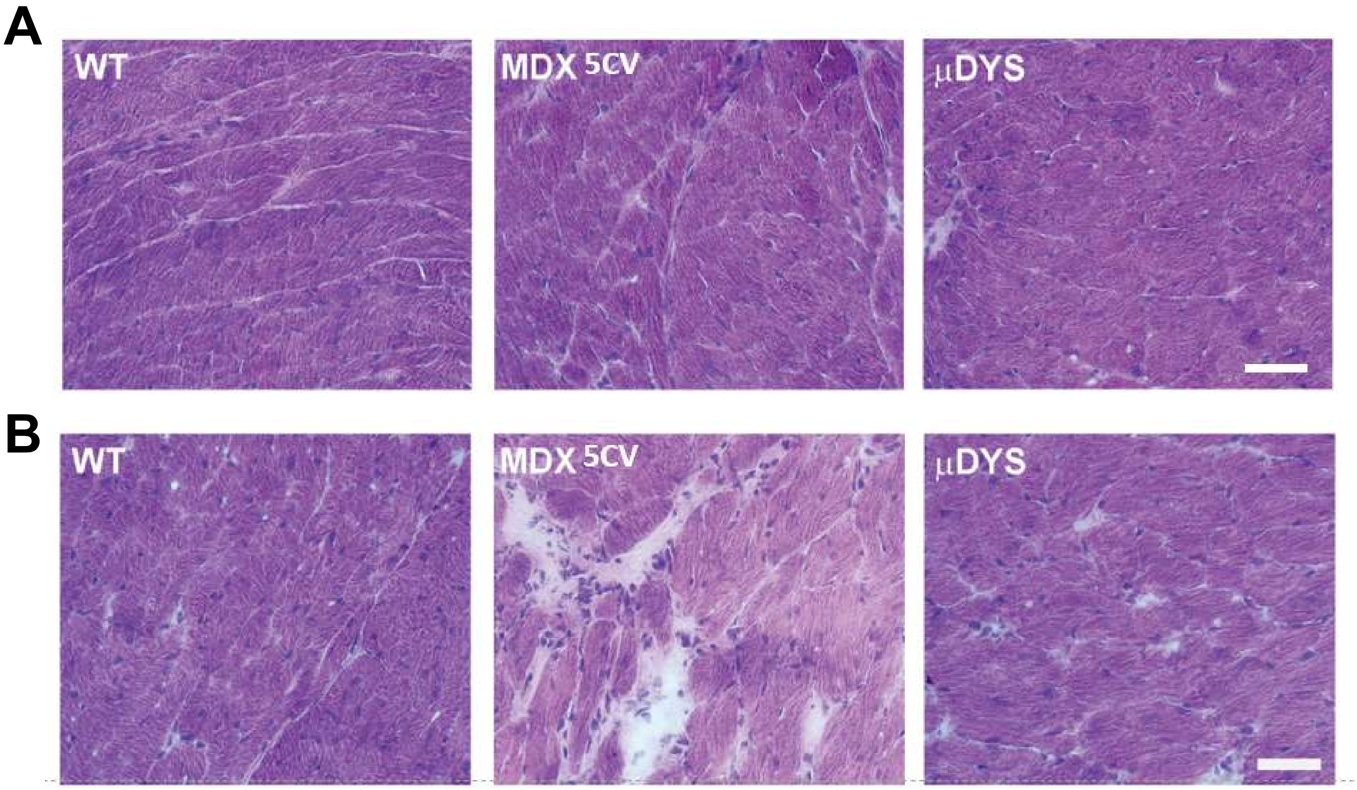

Figure S6: μDYS expression rescues cardiac histopathology in *mdx*^{5cv} mice. Representative pictures of Hematoxylin and Eosin stained heart sections from wild type (WT), *mdx*^{5cv} and μDYS-*mdx*^{5cv} mice (μDYS) at 6 months (**A**) and 1 year of age (**B**). Scale bars: 50μm.

Figure S7

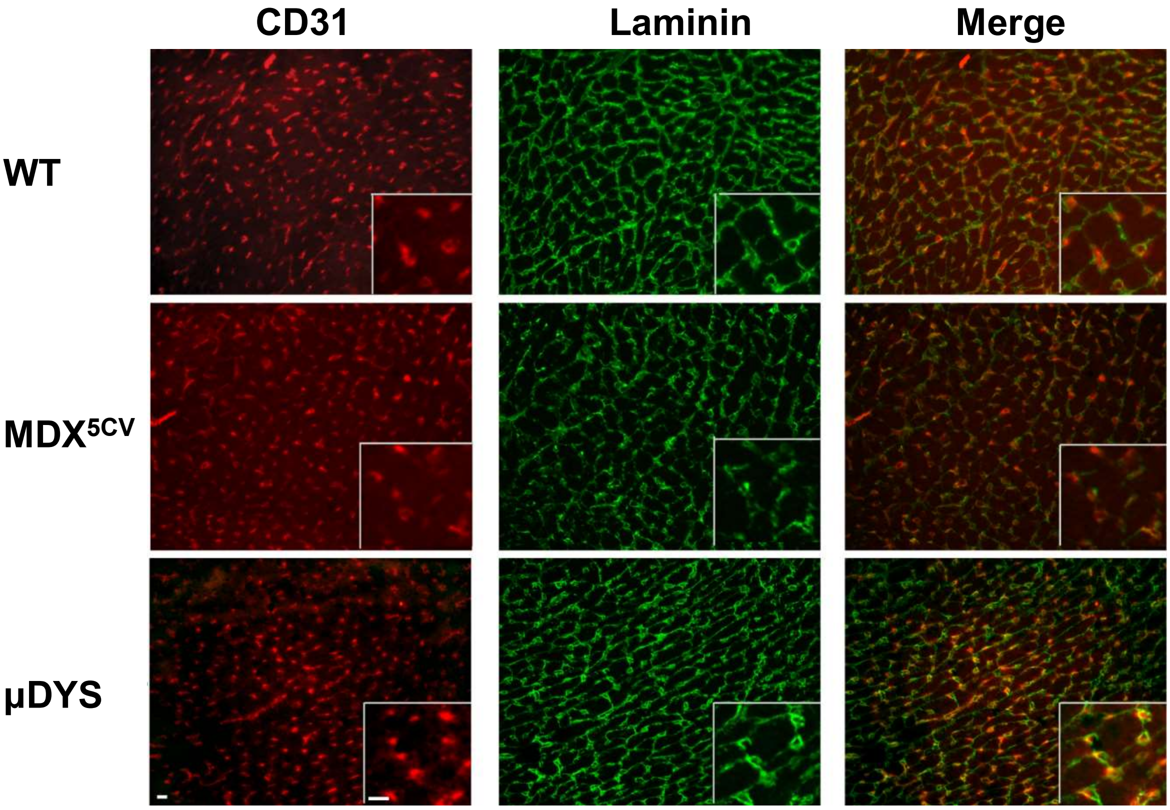

Figure S7: Immuno-labeling for quantification of capillary density and cardiomyocyte size. Representative pictures of heart sections from wild type (WT), *mdx*^{5cv} (MDX) and µDYS-*mdx*^{5cv} (µDYS) mice immunolabelled for CD31 (red) to visualize capillaries and a pan-laminin (green) to visualize boundaries of cardiomyocytes and blood vessels. Insets show magnifications to highlight capillaries recognizable by the expression of CD31 within a thin ring of laminin staining that is less than 10µm in diameter. Pictures shown are for mice at 6 months of age. Scale bars: 20µm. Uneven brightness of staining is due to the use of Sudan black to quench tissue auto-fluorescence. Images have been processed in Photoshop to increase contrast at the edges of the field and create a more even view of the staining. However, quantifications shown in Figure 1 were performed on unprocessed images.

Figure S8

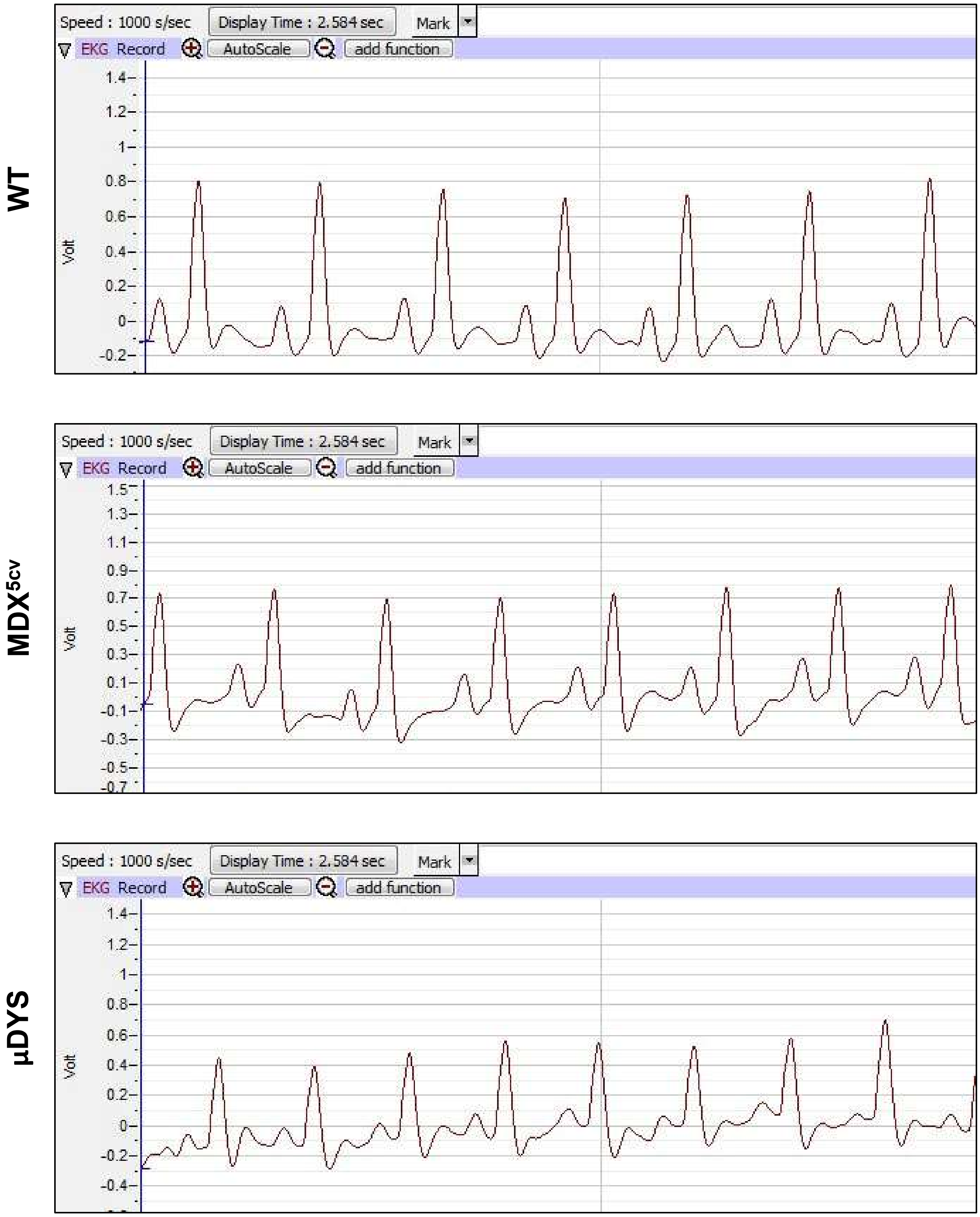

Figure S8: Representative electrocardiogram traces from wild-type, *mdx^{5cv}* and µDYS-*mdx^{5cv}* mice.

Figure S9

A

1 MEDVTLHIVE R**PYSGFPDAS** **SEGPEPTQGE** **ARATEEPSGT** **GSDELIKSDQ**
51 **VNGVLVLSLL** **DKIIGAVDQI** **QLTQAQLEER** **QAEMEGAVQS** **IQGELSK**LGK
101 **AHATTSNTVS** **K**LLEKVRKVS VNVKTVRGSL ERQAGQIK**KL** **EVNEAELLR**R
151 RNFK**VMIYQD** **EVK**LPAKLSV SKSLKESEAL PEKEGDELGE GERPEDDTAA
201 IELSSDEAVE VEEVIEESRA ERIKRSGLRR VDDFKKAFSK EKMEKTKVRT
251 RENLEKTRLK TKENLEKTRH TLEKRMNKLG TRLVPVERRE KLKTSRDKLR
301 **KSFTPDHVVY** **AR**SKTAVYK**V** **PPFTFHVK**KI **REGEVEVLKA** **TEMVEVGPED**
351 **DEVGAERGEA** **TDLLR**GSSPD VHTLLEITEE SDAVLVDKSD SD

B

1 MEDVTLHIVE RPYSGFPDAS SEGPEPTQGE ARATEEPSGT GSDELIK**SDQ**
51 **VNGVLVLSLL** **DKIIGAVDQI** **QLTQAQLEER** **QAEMEGAVQS** **IQGELSK**LGK
101 AHATTSNTVS KLLEKVRKVS VNVKTVRGSL ERQAGQIKKL EVNEAELLRR
151 RNFKVMIYQD EVKLPAKLSV SKSLKESEAL PEKEGDELGE GERPEDDTAA
201 IELSSDEAVE VEEVIEESRA ERIKRSGLRR VDDFKKAFSK EKMEKTKVRT
251 RENLEKTRLK TKENLEKTRH TLEKRMNKLG TRLVPVERRE KLKTSRDKLR
301 KSFTPDHVVY ARSKTAVYKV PPFTFHVKKI REGEVEVLKA TEMVEVGPED
351 DEVGAERGEA TDLLRGSSPD VHTLLEITEE SDAVLVDKSD SD

Figure S9: Cavin-1 peptides identified by proteomics. The full amino acid sequence coverage map of cavin-1 is shown. Amino acids in red correspond to peptides identified by proteomics in dystrophin immunoprecipitations from wild-type (**A**) and μDYS-mdx^{5cv} (**B**) mice.

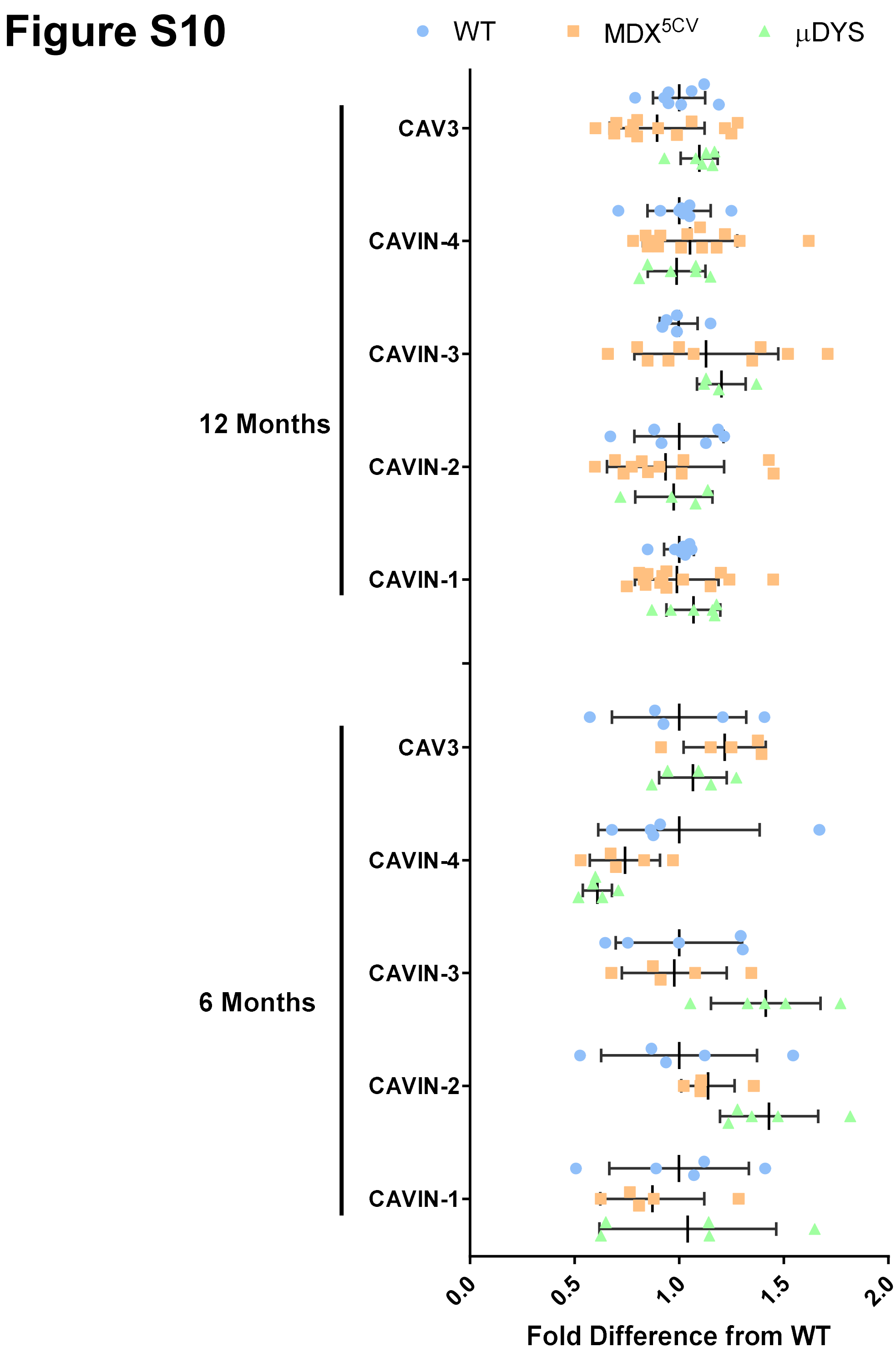

Figure S10: Expression levels of cavins and caveolin 3 in the heart of 6 and 12 months old wild-type, *mdx*^{5cv} and µDYS-*mdx*^{5cv} mice. Protein expression levels were quantified from western blots of total cardiac lysates probed with antibodies to cavins and caveolin-3 (Cav3) Protein levels were normalized to GAPDH probed on the same membrane. Data (mean ± standard deviations are expressed as fold differences relative to expression levels in wild-type lysates. No significant differences were found (one-way ANOVA).

Figure S11: Disruption of membrane localization of cavin-4 and cavin-2 in cardiomyocytes from *mdx^{5cv}* and µDYS-*mdx^{5cv}* mice. A. Representative high magnification pictures of tissue sections from of 6 months old wild-type (WT), *mdx^{5cv}* and µDYS-*mdx^{5cv}* (µDYS) mouse hearts double labelled with antibodies to cavin-2 (green) and laminin-α2 (red). Bright cavin-2 staining is observed in capillaries examples of which are indicated by asterisks. Arrows point to a cardiomyocyte membrane that is not adjacent to a capillary. Scale bar: 10µm. **B.** Representative low magnification micrographs of tissue sections of 6 months old wild-type (WT), *mdx^{5cv}* and µDYS-*mdx^{5cv}* (µDYS) mouse hearts double labelled with antibodies to cavin-4 (green) and dystrophin (red). Scale bar: 50µm.

Figure S11

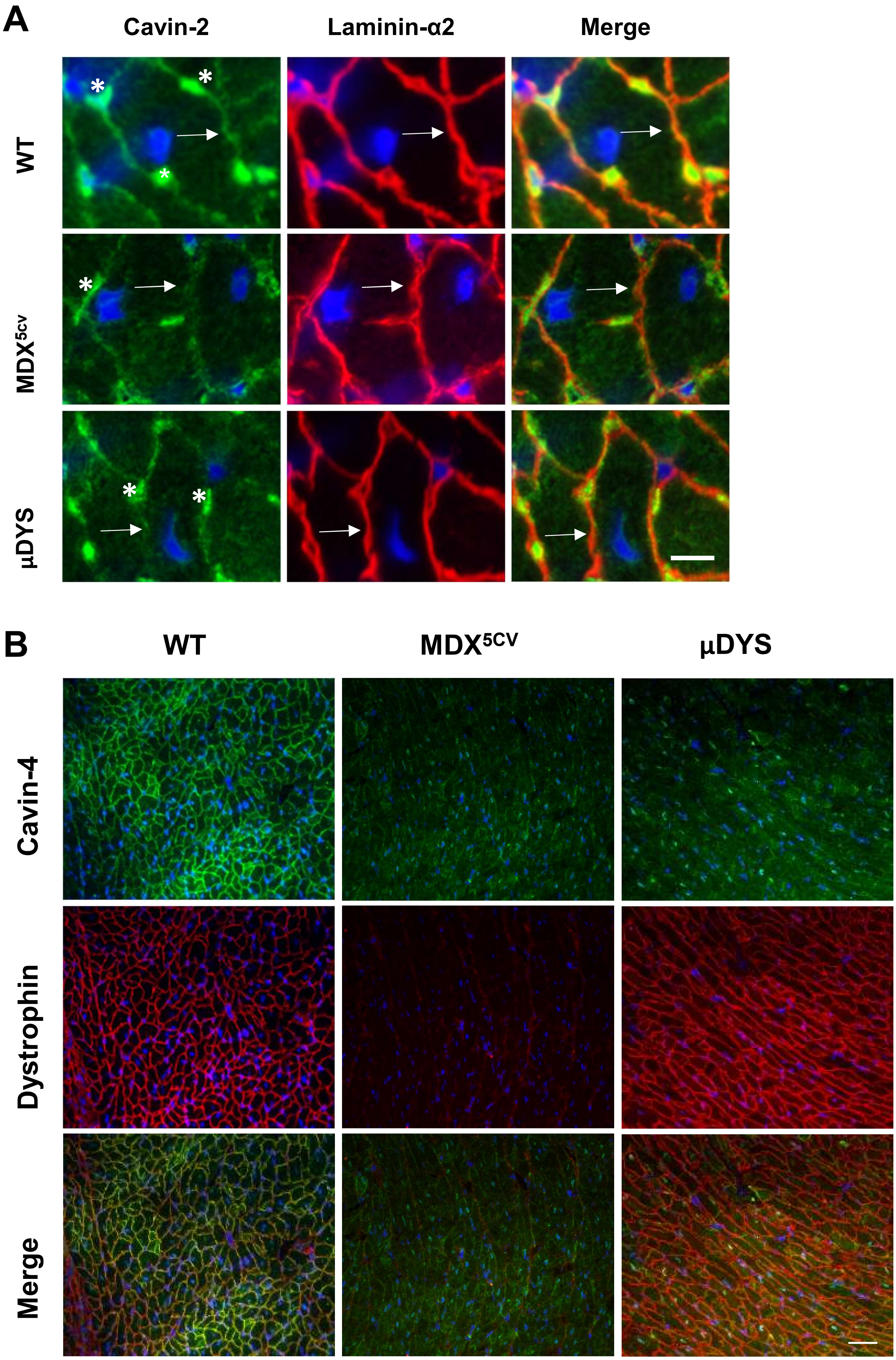

A
Cavin-2
Laminin-α2
Merge
WT
MDX5cv
µDYS
B
WT
MDX5CV
µDYS
Cavin-4
Dystrophin
Merge
