# Supplementary material for: Proteomic analysis identifies key differences in the cardiac interactomes of dystrophin and micro-dystrophin
Source: Hum Mol Genet. 2021 May 5;30(14):1321–36. doi: 10.1093/hmg/ddab133 (PMC8255133; doi:10.1093/hmg/ddab133)
Supplement: SUPPLEMENTAL_MATERIAL_Revised_Final_ddab133 [file supplemental_material_revised_final_ddab133.docx]

**SUPPLEMENTAL MATERIAL**

**DETAILED METHODS**

**Animals**

All experimental mice were bred in-house and kept under similar conditions. All mice were maintained in a specific-pathogen free animal care facility on a 12-hour light (25 lux):12-hour dark cycle with access to food and water ad libitum. Animal breeding and experimental procedures followed approved protocols by the Institutional Animal Care and Use Committees at Nationwide Children’s Hospital and University of Missouri.

Original C57BL/6J breeders were obtained from The Jackson Laboratory (Bar Harbor, ME). BL6Ros.Cg-*Dmd^mdx5cv^* (referred to as *mdx^5cv^*) breeders were kind a gift from Dr. L. Kunkel (Harvard Medical School, MA). Transgenic *mdx* mice expressing ΔR4-R23/ΔCT micro-dystrophin in cardiomyocytes were generated at the University of Missouri transgenic core. Briefly, the ΔR4-R23/ΔCT micro-dystrophin sequence was inserted between the α-myosin heavy chain (αMHC) promoter and the bovine growth hormone polyadenylation signal (BGH pA) in a multi-cloning vector (a gift from Dr. Jeffrey Robbins, Division of Molecular Cardiovascular Biology, Cincinnati Children’s Hospital Research Foundation, Cincinnati, Ohio) using standard molecular cloning procedures ^1^. The final construct was confirmed by sequencing cloning junctions and restriction analysis of the entire plasmid. A linear fragment of the full expression cassette (including the αMHC promoter, the microgene and the BGH pA) was then released and purified. Pronuclear injection, embryo culture and transplantation were performed on the FVB strain mouse by the University of Missouri Transgenic Core. The founder line DD529 was identified by PCR and it was originally backcrossed with C57BL10 background, dystrophin-null *mdx* mice for 5 to 7 generations to reach incipient congenic background status for C57BL10. Breeders were transferred to Nationwide Children’s Hospital to transfer the transgene onto the dystrophin deficient *mdx^5cv^* mouse strain (referred as μDYS-*mdx^5cv^*) for the purpose of proteomic studies. Compared to *mdx* mice, *mdx^5cv^* mice have a 10-fold lower frequency of revertant myofibers, the rare-occurring myofibers that spontaneously express an internally deleted dystrophin ^2^. Given the high sensitivity of the proteomics approach, *mdx^5cv^* mice are better suited to specifically study the interactome of micro-dystrophin. However, *mdx^5cv^* mice are on a different background strain (C57BL/6) than *mdx* mice (C57BL/10) and have more severe pathology ^3^. μDYS-*mdx^5cv^* mice used in these experiments have been backcrossed on the *mdx^5cv^* strain for up to 4 generations and are therefore on a mixed C57BL6 (93.75%) and C57BL10 (6.25%) background. Comparison of histopathological and electrocardiogram parameters were equivalent between *mdx* mice on a pure C57BL/10 background, *mdx^5cv^* mice on a pure C57BL/6 background, and transgene-negative *mdx^5cv^* mice on the hybrid background. No significant differences were found between male and female mice for any of the parameters studied here.

**Genotyping**

Transgenic *mdx* and *mdx^5cv^* micro-dystrophin mice were genotyped by PCR using the following primers to the transgene: DL254: 5’- GTCAGTGACCCAGAAGACGGAAGC-3’ and DL397: 5’- CTCATCCGTGGCCTCTTGAAGTTCC-3’. Reactions were multiplexed with the following internal control primers: Forward 5’-CAA ATG TTG CTT GTC TGG TG-3’, Reverse 5’-GTC AGT CGA GTG CAC AGT TT-3’. Control primers generated a band at 210 bp while the micro-dystrophin specific primers gave a band at 300 bp. Reaction conditions were as follows: 95^o^C for 10 min; then 25 cycles at 95^o^C for 30 sec, 61^o^C for 30 sec, 72^o^C for 1 min; followed by 72^o^C for 7 min. Reaction products were separated on a 1% agarose gel and ethidium bromide stained bands were visualized using a Gel Logic 200 Imaging System.

**Antibodies and antibody production**

Antibodies used for immunoprecipitations were generated in–house from hybridoma cells obtained from the Developmental Study Hybridoma Bank (Iowa). The MANEX1011B antibody against an epitope encoded by exons 10 and 11 of dystrophin was originally generated and extensively characterized by Glenn Morris ^4, 5^. The species and isotype (IgG2a) matched MW8 antibody against Huntingtin used as an immunoprecipitation control was originally developed by the Patterson lab ^6^. Hybridomas were grown in serum-free hybridoma medium (Invitrogen). The medium was collected after 10 days of culture and the antibodies were concentrated using a Centricon Plus-70 Ultracel PL-30 filter system (Millipore). Antibody concentration was determined by Western blot analysis at a range of dilutions against a purified mouse IgG antibody used as a standard.

Commercially available antibodies that had not been previously characterized were tested by Western blot for specific detection of band(s) at the expected molecular weight(s) and when possible by immunofluorescence (IF) on tissue sections for the expected labelling pattern. Antibodies satisfying these criteria are listed in the table below. References are provided for antibodies tested on tissues from knockout mice. Other antibodies tested that were found to recognize multiple bands or did not recognize a specific band at the correct molecular weight were: antibody AF5759 from R&D to cavin-2; antibody ab179923 from Abcam to cavin-3; antibodies PA5-25555 from Thermo Fisher and ab121644 from Abcam to cavin-4.

| Target protein | Vendor | Cat # | Host species | Dilution WB | Dilution IF |
| --- | --- | --- | --- | --- | --- |
| Cavin-1 *^7^* | Abcam | ab48824 | Rabbit | 1:1000 | 1:50 |
| cavin-1 (nt) | Abcam | ab88213 | Rabbit | 1:1000 | - |
| Cavin-2 *^7^* | Novus Biologicals | NBP1-44090 | Rabbit | 1:1000 | 1:50 |
| cavin-3 *^7^* | Abcam | ab83913 | Rabbit | 1:1000 | - |
| Cavin-4 | Abcam | ab121647 | Rabbit | 1:400 | 1:50 |
| Caveolin-3 *^8^* | BD Biosciences | BD 601420 | Mouse | 1:1000 | 1:50 |
| Laminin-α2 | Sigma | L0663 | Rat | - | 1:500 |
| laminin | Sigma | L9393 | Rabbit | - | 1:400 |
| collagen I | Abcam | ab292 | Rabbit | - | 1:50 |
| CD31 | BD Biosciences | clone 390 | Rat | - | 1:200 |
| Dystrophin | DSHB | MANEX1011B | Mouse | 1:100 | 1:200 |
| α1-syntrophin *^9^* | Abcam | ab11187 | Rabbit | 1:500 | 1:100 |
| b1-syntrophin *^10^* | Froehner Lab |  | Rabbit | 1:500 | - |
| β-dystroglycan *^11^* | DSHB | MANDAG2 | Mouse | 1:500 | 1:200 |
| a-Dystrobrevin | BD Biosciences | 610766 | Mouse | 1:500 | 1:200 |
| β-sarcoglycan | Abcam | ab83699 | Rabbit | 1:500 | 1:50 |
| ahnak-1 | Cedarlane | CLX332AP | Mouse | 1:1000 | - |
| ERK | Cell Signaling | 4696 | Mouse | 1:500 | - |
| phospho-erk | Cell Signaling | 9101S | Rabbit | 1:500 | - |
| cardiac troponin | Abcam | ab47003 | Rabbit | 1:400 | - |
| GAPdh | Millipore | MAB374 | Mouse | 1:5000 | - |

Secondary antibodies were purchased from Jackson ImmunoResearch Laboratories and had been serum pre-adsorbed for multi-labelling applications. They were used at a dilution of 1:200 for immunofluorescence and 1:5,000 for chemiluminescence in Western blot analyses. For LiCor applications, secondary antibodies conjugated to fluorophores of 700 or 800nm were purchased from Li-Cor Biosciences and diluted 1:10,000.

**Immunohistochemistry**

Hearts were mounted unfixed on oak chucks with the atria embedded in 7% tragacanth gum and flash frozen in isopentane cooled in liquid nitrogen. Tissues were cryosectioned and consecutive 5 μm sections were collected starting at the apex. For quantifications, we used sections at mid-level where both ventricles were visible. Unless otherwise specified below, immunolabelling was performed on non-fixed and non-permeabilized sections. Fresh tissue sections were blocked for 1 hour in PBS containing 5% horse serum. If a primary antibody made in mouse was used, sections were blocked for 1 hour in PBS containing 10% horse serum followed by a 2 hour incubation in PBS containing 0.1mg/ml Donkey-anti-mouse Fab fragments (Jackson ImmunoResearch Laboratories) to block endogenous immunoglobulins. Sections were single or double-labelled overnight at 4 ^o^C with primary antibodies then incubated for 1 hour with the appropriate secondary antibodies. Following immunolabelling, all tissue sections were counter-stained for 3 min with DAPI (1:10,000 dilution in water) to visualize nuclei then mounted in *n*-propyl-gallate mounting medium (<https://www.jacksonimmuno.com/technical/products/protocols/anti-fade>). Montages of entire sections were taken with a 10X objective on a fully motorized BX63 Olympus microscope equipped with a Hamamatsu Camera. Individual high-power pictures were taken as 1μm thick Z-stacks with a 40x objective. Images were flattened using the brightest pixel selection. All pictures were acquired with the same exposure settings to enable direct comparison of all genotypes.

For double labelling with antibodies to CD31 and laminin, the delicate punctate staining of capillaries surrounded by a laminin basement membrane was best visualized and quantified after quenching tissue auto-fluorescence. After incubation with secondary antibodies, tissue sections were fixed in 2% paraformaldehyde for 15 minutes, then incubated in a 0.7% Sudan black solution for 10 minutes prior to mounting.

**Quantification of capillary density and cardiomyocyte diameter**

Only photographs of cross-sectionally cut cardiomyocytes were chosen and were analysed blind. Capillaries were defined as CD31 positive staining surrounded by a laminin circular rim with a diameter less than 10μm. Capillaries were quantified from 3 pictures of the left ventricle per section per animal. Cardiomyocyte diameter was calculated based on laminin staining for a total of 300 cardiomyocytes per animal. The minimum Feret diameter was calculated using ImageJ. This parameter was chosen because it is not affected by the orientation of the cut and follows TREAT-NMD guidelines for the study of dystrophic muscles (DMD_M.1.2.001).

**Quantification of fibrosis**

All pictures were taken at the same exposure from sections that were all processed for immunostaining on the same day. Fibrosis was quantified on full montages of cardiac sections using the ImageJ software threshold function after having calibrated the image scale using a stage micrometer. A first threshold was set to completely outline the collagen-positive areas in an *mdx^5cv^* tissue section where pathological collagen I staining was present. This same threshold value was saved and then applied to all tissue sections from all genotypes to obtain a measurement of the fibrotic area in μm^2^. To calculate the total area of each tissue section, the threshold was increased until the entire section was outlined and a second measurement was taken to obtain the total section area. These values were used to calculate the percent collagen I positive area for each animal.

**Quantification of cavin-4 positive nuclei**

Tissue sections were triple-labelled for cavin-4, laminin α2 and DAPI. Laminin α2 staining only surrounds cardiomyocytes and was used to distinguish myonuclei from nuclei belonging to interstitial cells. Non-overlapping, randomly chosen fields (N=4) taken with a 20x objective were used to count the total number of myonuclei and the number of myonuclei positive for cavin-4. A minimum of 200 myonuclei were counted per animal using the counter tool in ImageJ.

**Quantification of membrane fluorescence intensity**

Measurements of immunofluorescence signal intensity at the lateral membranes of cardiomyocytes were performed as previously described ^12^. Tissue sections were double labelled for laminin α2 and the protein of interest. Pictures acquired with the same exposure settings using the MetaMorph software (Molecular Devices). Forty circular regions of interest with a diameter of 30 pixels were generated at random by the MetaMorph software and adjusted manually to overlap the sarcolemma and the adjacent cytoplasm of the nearest cardiomyocyte. For each region, the minimum fluorescence intensity measurement (background from the adjacent cytoplasm) was subtracted from the maximum fluorescence intensity measurement. Intensity measurements for the protein of interest were first normalized to intensity measurements taken for laminin α2 from the same picture to account for any regional and inter-image variation in fluorescence intensity or tissue thickness. Although laminin α2 is a ligand for α-dystroglycan, the intensity of laminin α2 staining at the basement membrane of cardiomyocytes showed no significant differences (two-way repeated measures ANOVA) among genotypes (means ± SEM are shown in orange):

**Electrocardiograms**

Mice were anesthetized under 3% isoflurane vaporized with 100% O2, and then maintained under 1% isoflurane anesthesia during data acquisition. Three ECG leads were placed on the upper left and right limbs and lower left limb, respectively.  After a 5 min equilibration period, ECGs were collected using a BioPac data acquisition system (Goleta, CA) and recorded over a 10 min period. Tracing were analyzed using LabScribe 2 ECG software (iWorx Systems, Inc, Dover, NH).  Data were analyzed for heart rate, Q-T interval duration, corrected Q-T interval duration, P-R interval duration, R wave amplitude, QRS duration and T wave amplitude. Data were collected from 10 different time points per animal over the 10 min recording period and then averaged.

**Immunoprecipitations**

Full-length dystrophin and ΔR4-R23/ΔCT micro-dystrophin were immunoprecipitated from fresh heart protein homogenates using the mouse monoclonal antibody MANEX1011B following the protocol we previously published for the anti-dystrophin antibody MANDYS1 ^13^. Heart homogenates for each mouse were kept separate and each immunoprecipitation represents an individual mouse. Control immunoprecipitations were performed on *mdx^5cv^* littermates that do not express the ΔR4-R23/ΔCT micro-dystrophin transgene using the MANEX1011B antibody or on wild-type mice the mouse monoclonal antibody MW8 to Huntingtin, a protein not expressed in heart tissues. Utrophin, a dystrophin homologue, was detected in some MANEX1011B immunoprecipitations (Table S1). No cardiac DAPC proteins were identified in control immunoprecipitations except for cypher and αB-crystallin that were present at very low levels in 3/5 control immunoprecipitations (Table S1) and were therefore excluded from all subsequent analyses.

**Liquid chromatography-tandem mass spectrometry (LC-MS/MS) and peptide sequence analysis**

Proteomic experiments involved immunoprecipitations from 4 wild-type, 4 *mdx^5cv^*, and 4 μDYS-*mdx^5cv^* mice using the MANEX1011B antibody and on 1 wild-type mouse using the MW8 antibody. LC-MS/MS and peptide sequence analysis were performed as previously described ^13^. Briefly, immunoprecipitated proteins were digested with trypsin overnight and peptides were analyzed by LC-MS/MS on a Thermo LTQ Orbitrap XL mass spectrometer (Thermo Fisher Scientific, San Jose CA) equipped with a microspray source (Michrom Bioresources Inc, Auburn, CA) operated in positive ion mode. Data was acquired with a spray voltage of 2.2 KV and a capillary temperature of 175 °C is used. The scan sequence of the mass spectrometer was based on the preview mode data dependent TopTen™ method: the analysis was programmed for a full scan recorded between *m/z* 350 – 2000 and a MS/MS scan to generate product ion spectra to determine amino acid sequence in consecutive scans of the ten most abundant peaks in the spectrum. To achieve high mass accuracy MS determination, the full scan was performed at FT mode and the resolution was set at 60,000. MS/MS was performed using ion trap mode to ensure the highest signal intensity of MS2 spectra. The CID fragmentation energy was set to 35%. Dynamic exclusion is enabled to facilitate tandem MS detection of lower abundant peptides. An exclusion list containing major trypsin autolysis peptides is applied so these peaks will not be detected. The reject mass width window is set at 30ppm.

The RAW data files were converted to mzXML and MGF files using MassMatrix data conversion tools (version 1.3, http://www.massmatrix.net/download). Resulting .mgf files were searched using Mascot Daemon (version 2.3.2, Matrix Science, Boston, MA) against Uniprot mouse database (version 12162011; 55,744 protein sequences). A customized database was constructed by replacing dystrophin with the μDys sequence to specifically identify and quantify the synthetic μDys. Trypsin was used as the enzyme and three missed cleavages were permitted. Considered variable modifications were oxidation (Met) and carbamidomethylation (Cys). The mass accuracy of the precursor ions was set to 10ppm and the fragment mass tolerance to 0.5 Da. Accidental picking of one 13C peak was included into the search. The significance identity threshold was set at p<0.05 for valid peptide and protein identification. False discovery rates (FDR) for peptide matches were estimated using the target-decoy search strategy ^14, 15^.

**Label free quantitation**

MS data were analysed in Scaffold (Proteome Software, Inc) with the following settings: 95% peptide threshold, 95% protein threshold and minimum of 3 peptides identified. Proteins identified in any of the 4 control immunoprecipitation samples or corresponding to immunoglobulins were excluded from analysis. The quantitative analysis comparing wild-type and μDYS-*mdx^5cv^* immunoprecipitations was performed on the proteins remaining after background subtraction. Label free quantitation was performed by calculating the Exponentially Modified Protein Abundance Index (emPAI) of each protein identified. This index is an exponential form of the number of observed peptides divided by the number of all possible tryptic peptides from a particular protein and provides an estimate of the abundance of each identified protein within a sample ^16, 17^. A two-tailed Student-t test with a significance threshold set at p<0.05 was performed on the emPAI values normalized to either the total spectra in each sample or to the emPAI of dystrophin/μDys within the same sample.

**Western blot analyses**

Ten to 15 cryosections of a 30μm thickness were collected from hearts of 6 and 12 months old wild type, *mdx^5cv^*, and μDYS-*mdx^5cv^* mice. Proteins were extracted for 10min on ice in lysis buffer containing 4% sodium dodecyl sulfate, 4M urea, 125mM Tris-HCL at pH 6.8, and a protease/phosphatase inhibitor cocktail (Pierce). The crude lysates were heated at 95°C for 3 minutes, then centrifuged at 14,000 rpm for 10 minutes at 4° (Eppendorf Centrifuge 5424R). Supernatant was collected and protein concentration was measured using the Pierce® BCA protein assay kit.

Samples were reconstituted in Laemmli reducing sample buffer 30μg protein were loaded per lane in NuPAGE™ bis-tris protein gels (Invitrogen). Gels were run in NuPAGE™ MOPS SDS running buffer for 1 hour at 200V. Proteins were then transferred for 1.5 hours at 30V onto a 0.45-micron nitrocellulose membrane. Membranes were stained in Ponceau S solution to verify the quality of protein transfer, and to mark the position of the lanes and molecular weight markers. To probe for multiple proteins of different molecular weight on the same samples, the membrane was cut into strips, thus avoiding possible artefacts introduced by membrane stripping. The nitrocellulose membrane was blocked with 5% milk in wash buffer (20mM Tris-HCL pH 7.5, 150mM NaCl, and 0.1% Tween-20) for one hour at room temperature. All primary antibodies were incubated overnight at 4°C in blocking solution containing either 5% milk or 5% bovine serum albumin based on prior antibody optimization tests. Membranes were incubated for 30 minutes at room temperature with the appropriate secondary antibody conjugated to HRP (1:5,000 dilution; Jackson ImmunoResearch, followed by incubation in enhanced chemiluminescence reagents (Supersignal™ West Pico PLUS Chemiluminescent Substrate, Thermo scientific). The signal was visualized using a ChemiDoc XRS+ machine (Bio-Rad). Alternatively, membranes were incubated with secondary antibodies conjugated to fluorophores of 700 or 800nm diluted 1:10,000 (Li-Cor Biosciences) for near infra-red imaging by an Odyssey imaging unit (Li-Cor Biosciences). Densitometry was performed on non-saturated images for samples on the same membrane using the Image™ Lab 6.0 Software (Bio-Rad) or Image Studio (Li-Cor Biosciences). Band intensities for the proteins of interest was normalized to the loading control (GAPDH or cardiac troponin depending on the protein molecular weight) probed on the same membrane. The relative band intensity was normalized to the wild type control samples run on the same membrane (4 wild type samples were run on each membrane).

**RNA analyses**

RNA was extracted from 30 ventricular tissue cryo-sections (20μm thick) in trizol with shaking. Samples were centrifuged to remove tissue debris and the supernatant mixed thoroughly with an equal volume of ethanol (95-100%). This mixture was then purified using the Direct-zol RNA kit (Zymo Research; R2062). RNA was eluted in nuclease free water. First strand cDNA was synthesized using the High-Capacity RNA-to-cDNA kit (Appliedbiosystems; 4387406) from 800ng of input RNA using oligo dT primers. For DAPC genes, validated real time assays were purchased from IDT with probes labelled with FAM: β-sarcoglycan (Mm.PT.58.1305984), β-dystroglycan (Mm.PT.58.5524327), α1-syntrophin (Mm.PT.58.32407120), α-dystrobrevin (Mm.PT.58.12547497). The DAPC real time assays were multiplexed with the mouse HPRT-VIC housekeeping gene assay from Thermo Fisher (Mm00446968_m1). Real time PCR was performed using Taqman Fast Advanced Master Mix (Life Technologies) using 10ul reaction volumes. Validation experiments were conducted confirming that dynamic range, efficiency, and limit of detection are unaffected in multiplexed compared to single target gene reactions. For genes associated with cardiac hypertrophy, quantitative RT-PCR was performed using the Sybr Green Master Mix and Gapdh as the housekeeping gene as previously described^18^. Primers used are: Nppa F-CGTGCCCCGACCCACGCCAGCATGGGCTCC and R-GGCTCCGAGGGCCAGCGAGCAGAGCCCTCA; Nppb F-AAGGGAGAATACGGCATCATT and R-ACAGCACCTTCAGGAGATCCA; Myh6 F-CAGAGATTTCTCCAACCCAGCTGCG and R-AGTCAGCCATCTGGGCGTCCG; Mhy7 F-AGCAGCAGTTGGATGAGCGACT and R-CCAGCTCCTCGATGCGTGCC; Gapdh F-CGTGCCGCCTGGAGAA and R-CCCTCAGATGCCTGCTTCAC. Each PCR primer set was validated to amplify with 90% or greater efficiency.

All reactions were run on an ABI OneStep thermocycler (Life Technologies). Gene expression fold change was determined by the delta-delta Ct method relative to Hprt or Gapdh as the housekeeping gene using the Data Assist software (Life Technologies).

**SUPPLEMENTAL MATERIAL REFERENCES**

1. Subramaniam A, Jones WK, Gulick J, Wert S, Neumann J and Robbins J. Tissue-specific regulation of the alpha-myosin heavy chain gene promoter in transgenic mice. *The Journal of biological chemistry*. 1991;266:24613-20.

2. Danko I, Chapman V and Wolff JA. The frequency of revertants in mdx mouse genetic models for Duchenne muscular dystrophy. *Pediatr Res*. 1992;32:128-31.

3. Beastrom N, Lu H, Macke A, Canan BD, Johnson EK, Penton CM, Kaspar BK, Rodino-Klapac LR, Zhou L, Janssen PM and Montanaro F. Mdx(5cv) mice manifest more severe muscle dysfunction and diaphragm force deficits than do mdx mice. *The American journal of pathology*. 2011;179:2464-74.

4. Bartlett RJ, Stockinger S, Denis MM, Bartlett WT, Inverardi L, Le TT, thi Man N, Morris GE, Bogan DJ, Metcalf-Bogan J and Kornegay JN. In vivo targeted repair of a point mutation in the canine dystrophin gene by a chimeric RNA/DNA oligonucleotide. *Nature biotechnology*. 2000;18:615-22.

5. Morris G, Man N and Sewry CA. Monitoring duchenne muscular dystrophy gene therapy with epitope-specific monoclonal antibodies. *Methods in molecular biology*. 2011;709:39-61.

6. Ko J, Ou S and Patterson PH. New anti-huntingtin monoclonal antibodies: implications for huntingtin conformation and its binding proteins. *Brain Res Bull*. 2001;56:319-29.

7. Hansen CG, Shvets E, Howard G, Riento K and Nichols BJ. Deletion of cavin genes reveals tissue-specific mechanisms for morphogenesis of endothelial caveolae. *Nature communications*. 2013;4:1831.

8. Woodman SE, Park DS, Cohen AW, Cheung MW, Chandra M, Shirani J, Tang B, Jelicks LA, Kitsis RN, Christ GJ, Factor SM, Tanowitz HB and Lisanti MP. Caveolin-3 knock-out mice develop a progressive cardiomyopathy and show hyperactivation of the p42/44 MAPK cascade. *The Journal of biological chemistry*. 2002;277:38988-97.

9. Eisinger K, Froehner SC, Adams ME, Krautbauer S and Buechler C. Evaluation of the specificity of four commercially available antibodies to alpha-syntrophin. *Anal Biochem*. 2015;484:99-101.

10. Kim MJ, Whitehead NP, Bible KL, Adams ME and Froehner SC. Mice lacking alpha-,beta1- and beta2-syntrophins exhibit diminished function and reduced dystrophin expression in both cardiac and skeletal muscle. *Human molecular genetics*. 2018.

11. Yoon JH, Johnson E, Xu R, Martin LT, Martin PT and Montanaro F. Comparative Proteomic Profiling of Dystroglycan-Associated Proteins in Wild Type, mdx, and Galgt2 Transgenic Mouse Skeletal Muscle. *J Proteome Res*. 2012;11:4413-24.

12. Arechavala-Gomeza V, Kinali M, Feng L, Brown SC, Sewry C, Morgan JE and Muntoni F. Immunohistological intensity measurements as a tool to assess sarcolemma-associated protein expression. *Neuropathol Appl Neurobiol*. 2010;36:265-74.

13. Johnson EK, Zhang L, Adams ME, Phillips A, Freitas MA, Froehner SC, Green-Church KB and Montanaro F. Proteomic analysis reveals new cardiac-specific dystrophin-associated proteins. *PloS one*. 2012;7:e43515.

14. Elias JE and Gygi SP. Target-decoy search strategy for mass spectrometry-based proteomics. *Methods in molecular biology*. 2010;604:55-71.

15. Elias JE and Gygi SP. Target-decoy search strategy for increased confidence in large-scale protein identifications by mass spectrometry. *Nat Methods*. 2007;4:207-14.

16. Ishihama Y, Oda Y, Tabata T, Sato T, Nagasu T, Rappsilber J and Mann M. Exponentially modified protein abundance index (emPAI) for estimation of absolute protein amount in proteomics by the number of sequenced peptides per protein. *Molecular & cellular proteomics : MCP*. 2005;4:1265-72.

17. Shinoda K, Tomita M and Ishihama Y. emPAI Calc--for the estimation of protein abundance from large-scale identification data by liquid chromatography-tandem mass spectrometry. *Bioinformatics*. 2010;26:576-7.

18. Brayson D, Frustaci A, Verardo R, Chimenti C, Russo MA, Hayward R, Ahmad S, Vizcay-Barrena G, Protti A, Zammit PS, dos Remedios CG, Ehler E, Shah AM and Shanahan CM. Prelamin A mediates myocardial inflammation in dilated and HIV-associated cardiomyopathies. *JCI Insight*. 2019;4.
